# Population dynamics analysis of an industrial methanotrophic consortium based on *Methylococcus capsulatus* KN2 using AI technologies

**DOI:** 10.3389/fmicb.2026.1831666

**Published:** 2026-06-17

**Authors:** Maksim V. Zakhartsev, Dmitriy A. Pavlov, Igor Y. Oshkin, Victoria A. Saltykova, Azret A. Kochkarov

**Affiliations:** 1 C1BioEngineering LLC, Moscow, Russia; 2 Research Center of Biotechnology RAS, Moscow, Russia; 3 Kuban State Agrarian University, Krasnodar, Russia

**Keywords:** methanotrophs, SCP, bacterial consortium, cell morphotypes, population dynamics, bioreactor

## Abstract

**Background/introduction:**

Monitoring the population dynamics of industrial methanotrophic bacterial consortia is critical for optimization of single-cell protein (SCP) production from natural gas. Traditional manual microscopy is labor-intensive, subjective, and limited in throughput. AI-based computer vision offers a promising alternative for automated, quantitative analysis of cell morphotypes in mixed cultures.

**Methods:**

A convolutional neural network (YOLO11x-seg) with a P2 activation layer for small-object detection and Focaler-MPDIoU loss function was trained on 250 phase-contrast micrographs of an industrial methanotrophic consortium based on *Methylococcus capsulatus* KN2, cultivated continuously at dilution rates of 0.15–0.25 h^–1^. The dataset comprised nine cellular morphotypes across 50,410 annotated objects (training: 200 images; validation: 50 images), with synthetic data augmentation applied to reduce class imbalance in the tetracocci class. The trained model was then applied to a pure *M. capsulatus* KN2 culture during substrate-unlimited batch growth (μ_max_ = 0.223 h^–1^, t_d_ = 3.11 h) across three biological replicates and four time points (3, 5, 7, 9 h), analyzing 277 micrographs and 15,124 objects.

**Results:**

On the validation set, the model achieved mAP@0.5:0.95 = 0.52, with class-weighted Precision = 0.87, Recall = 0.85, and F1 = 0.82. Per-morphotype F1 scores were: *monococci* 0.75, *diplococci*0.89, *tetracocci* 0.65. In the industrial consortium, producer cells (*M. capsulatus* KN2) constituted 88.5% of the population (*monococci* 33.7%, *diplococci* 61.9%, *tetracocci* 4.4%), while satellite bacteria comprised 11.5%. During batch cultivation of the pure producer strain, the *diplococci* fraction increased from 61.0% to 68.6% over 7 h, negatively correlating with a decline in *monococci* from 35.4% to 28.8% (Pearson *r* = –0.996, *p* = 0.004). *Tetracocci* showed no statistically significant correlation with either morphotype and are considered a stochastic subpopulation of *diplococci*. Cell cycle analysis revealed elongation of the M-phase from 71.6% to 78.4% of t_d_. *Monococci* cell radius, intracellular volume, and periplasmic surface area all increased over 9 h, while the surface-to-volume ratio declined.

**Discussion/conclusion:**

The observed M-phase elongation is consistent with incipient substrate limitation (CH_4_ or O_2_) in gas-tight batch flasks, detectable through morphotype ratio shifts before standard process parameters register any change. The approach enables a proof-of-concenpt for real-time culture quality monitoring, early prediction of growth limitations, and optimization of SCP production in industrial bioreactors.

## 1 Introduction

A promising approach for single-cell protein production involves the cultivation of a methane-oxidizing bacterial consortium on natural gas. This consortium is dominated by obligate aerobic methanotrophic bacteria that utilize methane as their sole source of carbon and energy. The resulting product, obtained as dried and inactivated biomass, is classified as bacterial single-cell protein (SCP). It is intended to balance and enrich the protein content of diets for livestock, poultry, fish, and aquaculture species.

The chemical composition of bacterial SCP produced from natural gas (i.e., gaprin) is characterized by a high crude protein content, reaching up to 75%, a substantial fraction of which consists of short-chain, water-soluble proteins that are readily digestible by animal organisms. The amino acid profile of methane-derived SCP is well balanced and distinguished by a high content of essential amino acids, including lysine, methionine, cysteine and tryptophan (Ritala et al., 2017; Zhuang et al., 2024). In addition, natural gas–derived SCP contains significant amounts of B-group vitamins (Bratosin et al., 2021). In terms of protein content and amino acid composition, SCP produced from natural gas outperforms several conventional feed protein sources, such as soybean meal (up to 45% protein) and feed yeast (up to 40% protein). Moreover, with respect to amino acid balance and bioavailability, gaprin is comparable to a fish meal, which typically contains up to 65% protein. Overall, natural gas-derived SCP represents a promising, high-quality source of feed protein that combines high nutritional value with biotechnological efficiency and broad application potential (European Commission, 1995; European Commission, 2011).

Industrial SCP production from natural gas relies predominantly on bacteria of the genus *Methylococcus*, which belong to type I methanotrophs and are affiliated with the class Gammaproteobacteria and the family *Methylococcaceae*. *Methylococcus* species are characterized as Gram-negative, non-motile, obligate aerobes with an optimal growth temperature of approximately 40–45°C (Bowman, 2015). They possess both membrane-bound (EC 1.14.18.3) and soluble (EC 1.14.13.25) forms of methane monooxygenase (MMO), which catalyze the oxidation of methane to methanol (Rosenzweig, 2015), which is assimilated through anabolic processes. Carbon assimilation proceeds via the ribulose monophosphate (RuMP) pathway, in combination with a non-canonical Calvin cycle and the serine pathway (Dworkin and Foster, 1958; Ward et al., 2004). *Methylococcus* strains have been isolated from a range of habitats, including soils, wastewater sludge, river and pond sediments (Bowman, 2015; Awala et al., 2020; Awala et al., 2021; Oshkin et al., 2021).

**FIGURE 1:**
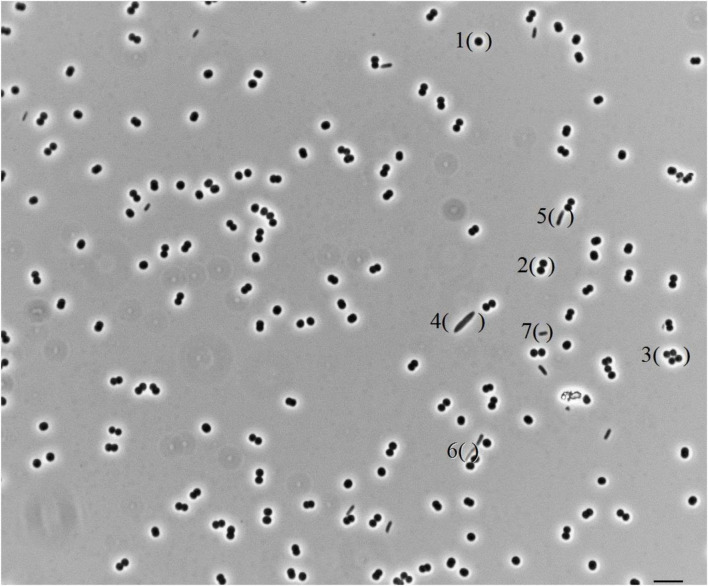
Representative micrograph of a mixed methanotrophic industrial consortium based on the producer strain *Methylococcus capsulatus* KN2. Producer cells are represented by morphotypes *monococci* (1), *diplococci* (2), and *tetracocci* (3); satellite bacteria by bacilli-like (4), long rods (5), thin rods (6), small rods (7), arc cells, and double cells (for more descriptions see Supplementary Table 1). Species names of satellite bacteria constitute proprietary know-how of the gaprin production technology developers and are therefore not disclosed. Scale bar: 5 μm.

In industrial processes, *Methylococcus* strains are cultivated as part of a complex, multicomponent microbial consortium that includes heterotrophic satellite bacteria (Bothe et al., 2002; Oshkin et al., 2020). As a rule, industrial production communities are formed purposefully (not randomly) through long-term co-cultivation of the producer and satellites strains. Microscopic analysis of these methanotrophic consortia reveals that both the primary producer and the satellite bacteria exist as diverse cell morphotypes (Figure 1). The precise taxonomic composition of the satellite bacteria, as well as the procedures used to establish an active industrial methanotrophic consortium constitutes proprietary know-how and is typically not disclosed. Nevertheless, the major morphotypes of satellite species observed in this study belonged to non-pathogenic aerobic bacteria, predominantly represented by the families *Brevibacillaceae*, *Paenibacillaceae*, and *Burkholderiaceae*.

Satellite bacteria utilize intermediate and end metabolic products of the primary methanotrophic producer (e.g., organic acids, alcohols, etc.), thereby preventing their accumulation in the culture medium and alleviating metabolic inhibition of consortium growth (Kalyuzhnaya et al., 2013; Jang et al., 2023). Consequently, the use of a complex bacterial consortium (producer: satellites) helps to achieve both the (i) higher biomass concentration (*C_x_*) and (ii) increased process productivity (*Q_x_*) (provided that the mass transfer characteristics of the cultivation apparatus allow for a high growth rate of high biomass concentration) (Petersen et al., 2017). This, in turn, (iii) allows SCP production from natural gas to be conducted in continuous-flow processes under non-sterile conditions, (iv) enabling prolonged operation / extended run times. These factors collectively lead to a substantial reduction in operational costs.

The economics of industrial-scale, high-productivity SCP production are based on long-term continuous cultivation of a mixed methanotrophic bacterial consortium characterized by high biomass concentrations and rapid growth rates under non-sterile conditions. The relative abundance of bacterial species and their cell morphotypes within the consortium is a key quality parameter that reflects the physiological state of the microbial community and determines the nutritional value, safety, and efficiency of gaprin utilization in livestock and aquaculture diets. Therefore, ensuring consistently high and reproducible product quality requires maintaining strict control over the culture’s stability throughout cultivation, including its taxonomic composition, producer-to-satellite ratio, and *monococci*:*diplococci* ratio.

**FIGURE 2:**
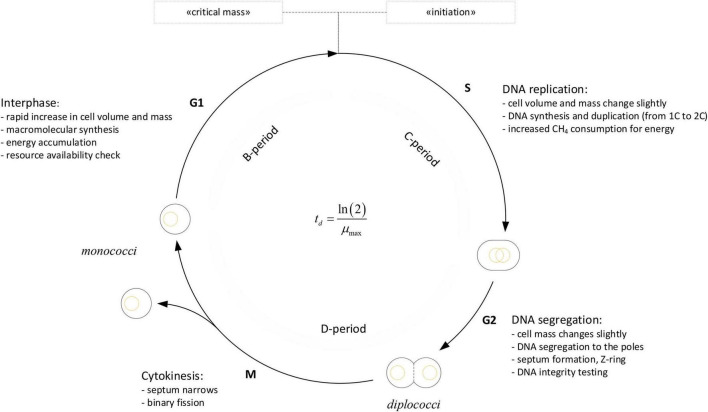
The cell cycle of *Methylococcus capsulatus*. The *tetracocci* morphotype is not shown in the diagram, as it is a subpopulation of the *diplococci* morphotype.

Under optimal conditions in an actively growing industrial consortium based on *Methylococcus capsulatus*, methanotrophic cells typically account for approximately 90% of the total biomass (considering all cell morphotypes), while the remaining ∼10% is represented by satellite bacteria (Zhuang et al., 2024; Bothe et al., 2002). Under conditions of steady-state growth with a known dominance of the producer strain and its morphotype distribution (*monococci*, *diplococci*, and *tetracocci*), the duration of specific cell cycle phases can be estimated (Figure 2). Temporal changes in the relative duration of these cell cycle phases provide insight into emerging factors that may limit biomass growth.

Accordingly, monitoring the *monococci*: *diplococci* (+*tetracocci*) morphotype ratio in *Methylococcus capsulatus* cultures can serve as a predictive tool for detecting and anticipating the onset of growth-rate limitation. For example, during the B period (i.e., analog of the G1 phase), *monococci* assess intracellular energy reserves and resource availability prior to entering the C period (i.e., analog of the S phase) (Wang and Levin, 2009). Under resource-limited conditions, the proportion of *monococci* increases relative to *diplococci*. Conversely, sufficient resource availability and energy accumulation promote an increase in *diplococci*, as the cytokinesis-associated D period (i.e., analog of the G2 and M phases) becomes longer than the combined B and C periods (Figure 2). As a reference benchmark, the *monococci*-to-*diplococci* (and *tetracocci*) ratio can be determined from substrate-unlimited batch cultures of pure *Methylococcus capsulatus* growing at their maximum specific growth rate (μ_max_).

The taxonomic composition and relative abundance of microorganisms in a complex culture (i.e., consortium) can be assessed using various analytical methods (Supplementary Table 2). The traditional microscopy approach, which involves manual cell counting from micrographs combined with consortium morphometric analysis, is labor-intensive and inherently subjective. Depending on cell density, an experienced technician typically requires up to 1 h to analyze a single image. To intensify data acquisition and improve result reliability, it is necessary to (i) increase the frequency of analysis, (ii) expand the volume of analyzed data, and (iii) eliminate subjectivity in data interpretation. To address the latter challenge, the application of computer vision methods and artificial intelligence technologies is particularly promising. For example, convolutional neural networks (CNNs) can be employed to automatically recognize and segment bacterial morphotypes in images acquired by light phase-contrast microscopy. CNNs are a specialized class of artificial neural network architectures designed for processing spatially structured data such as images. This integrated approach enables automated classification of cells by morphotype and supports accurate morphometric analysis, including calculation of the area of each identified object. In turn, this allows quantitative, real-time assessment of bacterial population dynamics within a microbial consortium (when combined with flow-through microscopy), which represents a critical factor for cultivation process optimization and quality control of the final product (SCP).

Thus, the objective of this study is to develop a neural network-based computer vision module for the acquisition of statistical data on the structure, parameters, and dynamics of a methanotrophic consortium based on *Methylococcus capsulatus* cultivated in a bioreactor. First, the microphotography of mixed industrial methanotrophic consortium (Figure 1) where all cellular morphotypes are presented, was grown in continuous cultivation mode and used to collect stable datasets suitable to train AI. Second, to modulate the cellular dynamics in the culture population, the pure methanotroph culture (only producer without satellites) was grown in substrate-unlimited batch mode in the exponential phase.

The developed tool is intended to perform the following tasks:

(1) automated recognition (detection) of cellular morphotypes and quantification of their abundance within the optical field, as morphotype ratios serve as indicators of culture quality (producer-to-satellite composition), while temporal changes in consortium structure reflect different phases of the producer strain cell cycle;
(2) calculation of intracellular volume (μm^3^ cell^–1^) and cell surface area (μm^2^ cell^–1^) for each cell class, parameters that are directly associated with metabolic fluxes of individual components measured in the bioreactor [g_*i*_ (g_*x*_⋅h)^–1^].

## 2 Materials and methods

### 2.1 Strain and cultivation conditions

Experiments were performed using *Methylococcus capsulatus* strain KN2 (Oshkin et al., 2021). The strain was cultivated on modified version of nitrate mineral salts (mNMS) medium containing (per liter of distilled water, g L^–1^) KNO3–0.2; MgSO_4_ × 7H_2_O–0.2; CaCl2 × 2H_2_O–0.04; and 0.1% (v/v.) of a trace element solution composed of (g L^–1^): EDTA,–5.0; FeSO_4_ × 7H_2_O–2.0; ZnSO4 × 7H_2_O–0.1; MnCl2 × 4H_2_O–0.03; CoCl2 × 6H_2_O–0.2; CuSO4 × 5H_2_O–0.1; NiCl2 × 6H_2_O–0.02; Na2MoO4–0.03. After sterilization, sterile phosphate buffer (a mixture of 18.9 g L^–1^ KH2PO_4_ and 9.6 g L^–1^ Na2HPO_4_ × 2H_2_O; pH 6.3) was added to the medium cooled to 60°C at a ratio of 1:100. Cultivation was carried out in sealed 500 mL flasks containing 50 mL of liquid mineral medium. Inoculated flasks were sealed with gas-tight rubber septa. Methane was added aseptically using a syringe equipped with a disposable 0.22 μm filter until a final concentration of 30% (v/v) in the gas phase was achieved. The flasks were incubated on an orbital shaker at 150 rpm and 42°C for 24 h. During cultivation, the gas phase was not refilled.

### 2.2 Microscopy and image acquisition

Morphophysiological characteristics of the culture were examined using Zeiss Axioplan 2 microscope equipped with Axiovision 4.2 software (Zeiss, Germany) at × 1,000 magnification (10 × 100) with immersion oil.

To obtain high-quality micrographs, samples were concentrated prior to slide preparation. From each flask, 1 mL of culture was collected, cells were pelleted by centrifugation at 8,000 × g for 2 min, and the pellet was resuspended in 50 μL of mNMS medium. The resulting suspension was placed on a glass slide and examined microscopically. Images were saved in JPG format at a resolution of 1,292 × 968 pixels. For each experimental flask and each time point, at least 15 micrographs were acquired (Table 1). The steps of culture concentration and coverslip fixation introduce inherent variability, which precludes the use of absolute cell counts per microscopic field as a reliable parameter for analyzing population growth dynamics. Therefore, micrograph analysis was used exclusively to assess relative morphotype abundances (f_*i*_), while the number of identified objects was applied only to evaluate the density (i.e., normality) of parameter distributions.

**TABLE 1:** Number of identified cells morphotypes *n* (*monococci, diplococci, tetracocci*) on *m* analyzed images collected during the substrate-unlimited batch growth of the pure *Methylococcus capsulatus* KN2 culture.

Cultivation time, [h]	Cell morphotypes	Flask 1		Flask 2		Flask 3		Total	
		*n*	*m*	*n*	*m*	*n*	*m*	*n*	*m*
3	*Monococci*	143	15	388	26	383	21	914	62
	*Diplococci*	231		747		596		1,574	
	*Tetracocci*	18		34		40		92	
	Total	392		1,169		1,019		2,580	
5	*Monococci*	410	23	427	19	287	22	1,124	64
	*Diplococci*	829		808		559		2,96	
	*Tetracocci*	41		48		33		122	
	Total	1,280		1,283		879		3,442	
7	*Monococci*	347	16	298	17	456	17	1,101	50
	*Diplococci*	854		637		1,281		2,772	
	*Tetracocci*	53		38		72		163	
	Total	1,254		973		1,809		4,036	
9	*Monococci*	545	17 71	436	16 78	481	18 78	1,462	51 277
	*Diplococci*	1,112		898		1,367		3,377	
	*Tetracocci*	92		49		86		227	
	Total	1,749		1,383		1,934		5,066	
	Total	4,675		4,808		5,641		15,124	

### 2.3 Measurement of biomass growth parameters in substrate-unlimited batch culture of Mc. capsulatus KN2

**FIGURE 3:**
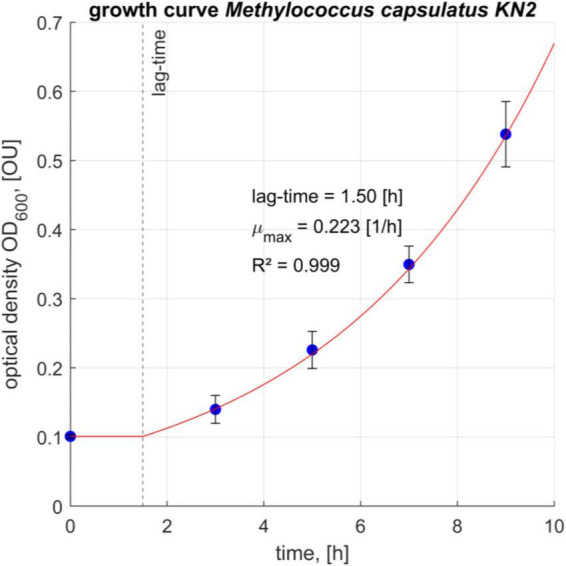
Change in optical density *OD*_600_ of the pure *Methylococcus capsulatus* KN2 culture in the substrate-unlimited batch experiment (*average* ± *sd*, *n* = 3). After the lag-phase (1.50 [h] with μ_*max*_ = 0 [1/h]) the culture grew at a stationary maximum specific growth rate μ_*max*_ = 0.223 [1/h] [cell doubling time *t_d_* = 3.11 [h], Equation (2)] throughout the entire early exponential growth phase of the cultivation.

**FIGURE 4:**
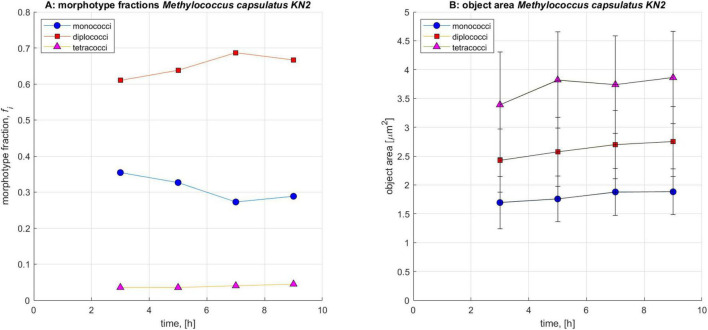
Dynamics of changes in **(A)** the fractional composition of the culture (f_*i*_) and **(B)** the calculated “shadow” area ( ± sd) of cell morphotypes [μm^2^] for the pure culture *Methylococcus capsulatus* KN2 during substrate-unlimited batch growth with μ_*max*_ = 0.223 [1/h]. The experimental data are provided in Table 4.

Optical density was measured at 600 nm (OD600) using a Spectroquant Prove 300 spectrophotometer (Merck, Germany). The inoculum of *Mc. capsulatus* KN2 was prepared by cultivation for 24 h under the conditions described above. Actively growing cultures were transferred into three identical flasks containing 50 mL of mNMS medium. To ensure exponential growth, the initial optical density in each flask was adjusted to OD600 = 0.1 (Figure 3). This low initial biomass concentration did not provide sufficient statistical material for morphotype analysis at t = 0. Therefore, data for this time point are absent in Figure 4. Cultivation was subsequently carried out according to the standard protocol. For OD600 measurements and microscopy, 1 mL samples were withdrawn aseptically through the rubber septum using sterile syringes. The first measurement was performed after 3 h, followed by subsequent measurements at 2 h intervals (Figures 3, 4).

Mean OD600(t) values averaged over three biological replicates (Figure 3) were analyzed using an ordinary differential equation (ODE) describing exponential microbial growth with inclusion of a *lag* phase. The model is given as:


$$\frac{d\,O\,D_{600}}{d\,t}=\mu_{\max}\times O\,D_{600}\,\left(t\right)$$ (1)

where:

1. OD_600_(t)–denotes the optical density of the culture [OD units] at time *t* and represents the state variable of the ODE model;

2. μ_max_–is the maximum specific biomass growth rate [1/h], estimated via parametric identification;

3. OD_600_ = 0.1 is the experimentally measured initial optical density (Figure 3), used as the initial condition of the ODE model;

During the *lag* phase, the model assumes the absence of growth [i.e., μ_max_ = 0 (1/h)]. The *lag*-phase duration (*lag* time) was estimated as part of the parameter optimization procedure.

Parameter estimation for μ_*max*_ and *lag* time was performed using a pattern-search optimization algorithm implemented in MATLAB. The objective function was defined as maximization of the coefficient of determination (*R*^2^) between experimental and model-predicted OD_600_(t) values. This approach provides quantitative validation of growth kinetics and enables robust estimation of key parameters for subsequent analyses.

### 2.4 Development and training of a neural network for cell morphotype recognition

A dataset comprising 250 microscopy images of a mixed methanotrophic culture (i.e., consortium) based on *M. capsulatus* KN2 was used to train the neural network. Images were obtained during preliminary continuous-cultivation experiments conducted in a bioreactor at dilution rates of D = 0.15–0.25 h^–1^.

Manual image annotation was performed using CVAT (Computer Vision Annotation Tool), an open-source platform for image labeling in computer vision applications.^1^ Using CVAT, a segmentation dataset with nine object classes was generated (Table 2).

**TABLE 2:** Statistics on cell morphotype classes in the mixed methanotrophic culture (i.e., consortium) based on *Methylococcus capsulatus* KN2, manually annotated on 250 experimental images obtained in continuous cultivations using the CVAT platform and used in the Training dataset (*m* = 200 images) expanded with the Synthetic dataset (*m* = 12 images) and the Validation dataset (*m* = 50 images) for neural network training.

Class	Category	Cell morphotype	Number of objects in the training dataset	Number of objects in the synthetic dataset	Number of objects in the validation dataset	Total	Fraction (*f_i_*)*	Average (median) “shadow” area of the object * [*μm^2^*] ± *s.d.*
0	Producer	*Diplococci*	22,053		5,590	27,643	0.548	2.57 (2.50) ± 0.65
1	Producer	*Tetracocci*	1,737	692	240	1,977 + 692 = 2,669	0.039	3.69 (3.50) ± 1.26
2	Producer	*Monococci*	11,764		3,239	15,003	0.298	1.84 (1.81) ± 0.39
3	Satellite	*Long_rods*	1,152		597	1,749	0.035	1.90 (1.72) ± 0.95
4	Satellite	*Arc_cells*	29		9	38	0.001	1.18 (1.09) ± 0.45
5	Satellite	*Double_cells*	177		21	198	0.004	2.59 (2.05) ± 1.40
6	Satellite	*Small_rods*	1,853		515	2,368	0.047	0.959 (0.911) ± 0.368
7	Satellite	*Thin_rod*	504		139	643	0.013	0.965 (0.876) ± 0.479
8	Satellite	*Bacilli-like*	631		160	791	0.016	3.16 (3.06) ± 1.13
**Total (with synthetic dataset)**			**39,900**	**692**	**10,510**	**51,102**		
**Total (without synthetic dataset)**			**39,900**		**10,510**	**50,410**	**1**	
**Number of annotated photographs**			**200**	**12**	**50**	**262**		

* Data for cell morphotypes in the original experimental dataset, excluding the synthetic dataset.

The annotated dataset was split into training (80%, *m* = 200 images) and validation (20%, *m* = 50 images) subsets, ensuring representative sampling. In total, 50,410 objects were annotated, including 39,900 objects in the training set (79.2%) and 10,510 objects in the validation set (20.8%) (Table 2).

The original dataset contained an insufficient number of *tetracocci*, resulting in class imbalance, which can negatively affect model performance. Class imbalance may (i) bias the model toward more frequent classes, (ii) lead to underfitting for rare classes, and (iii) influence optimal classification thresholds, thereby reducing overall model accuracy.

To address this issue, synthetic data augmentation was applied to increase the representation of rare classes. Since accurate identification of *monococci*, *diplococci*, and *tetracocci* is critical for assessing morphotype ratios, and since *tetracocci* occur infrequently, a synthetic dataset was generated specifically for the tetracoccus morphotype of the producer strain KN2.

#### 2.4.1 Generation of synthetic dataset

Synthetic data were generated to (i) address class imbalance by augmenting the *tetracoccus* class and (ii) create representative projections of *tetracoccus* cell morphology. A three-dimensional geometric model of a *tetracoccus* was constructed using Python and the Matplotlib library with the mplot3d toolkit.

**FIGURE 5:**
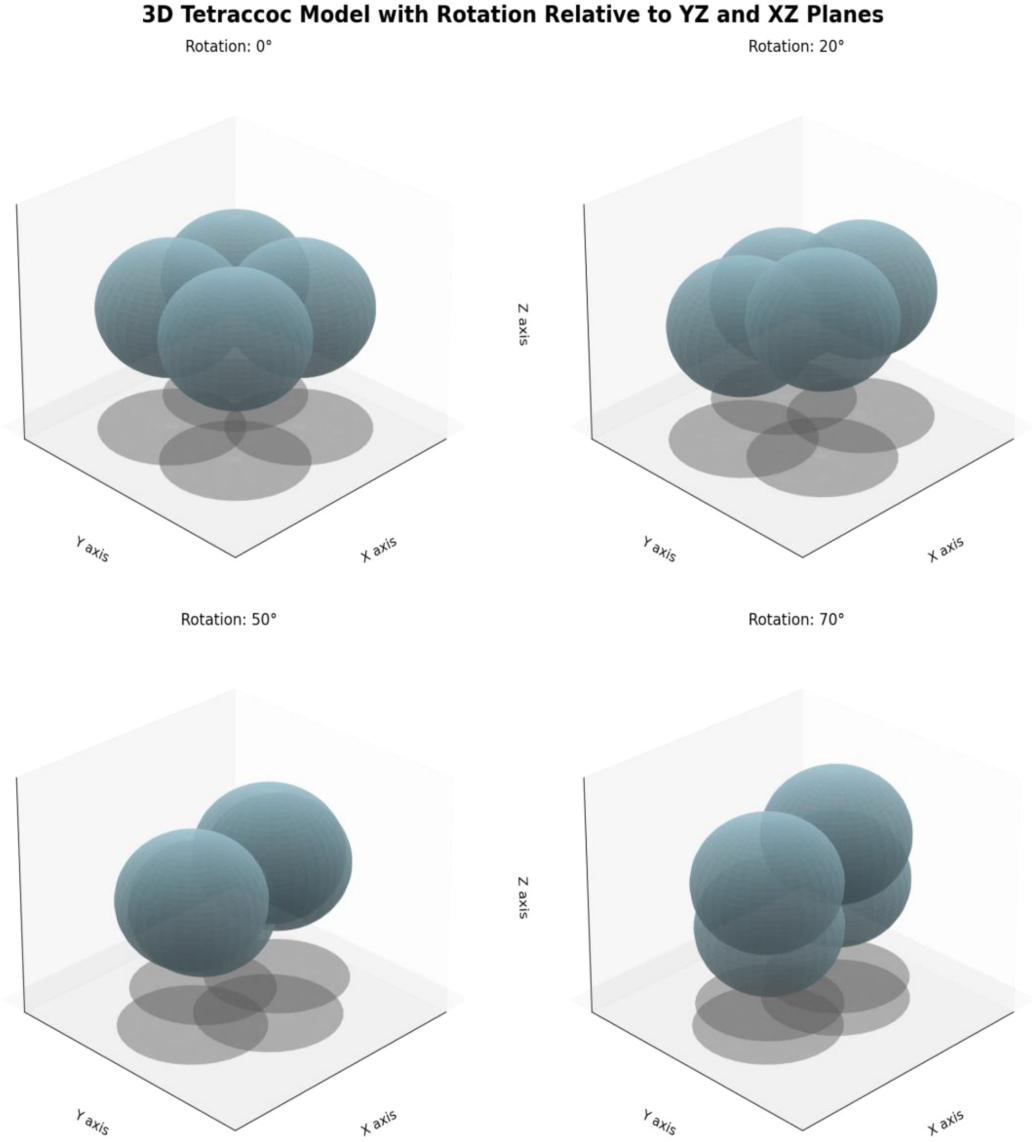
A 3D model of a tetracoccus with the object’s shadow projected onto the XY-plane. A synthetic dataset comprising 12 images with a total of 692 objects was generated based on these projections. Figure 7 presents an example of a synthetic image containing various *tetracocci* projections. The generated images were added to the Train dataset and annotated using the CVAT software. Consequently, the train dataset was expanded from m = 200 to m = 212 images, containing 692 additional *tetracocci* (see Figure 8), and was subsequently used for neural network training.

The model consisted of four identical spherical *monococci* (radius *r* = 0.75 μm) arranged in a single plane with 20% overlap. Two-dimensional projections (“shadows”) of the *tetracocci* were generated by rotating the 3D object in 5° increments relative to the image plane. Figure 5 illustrates the 3D tetracoccus model and representative projections obtained during rotation about the YZ and XZ planes.

#### 2.4.2 Cell morphotype segmentation and detection

Cell segmentation was performed using the YOLO11-seg architecture with a pretrained YOLO11x-seg model (62.1 million parameters).^2^ The model incorporates default multiple data augmentation techniques including: flipping, rotation, translation, brightness and contrast adjustment, mosaic augmentation, cropping and padding, blurring, noise injection, and HSV augmentation. These augmentations were applied during training to improve model robustness to variations in illumination, scale, orientation, and background complexity.

#### 2.4.3 Training of a neural network

The design, development and training procedure of the neural network (e.g., Model architecture and loss function, Weighted loss function to reduce class imbalance, P2-layer activation; Two-stage training; Training hyperparameters; Training metrics) are described in details in Supplementary material 3 in order to save the space in a biological text.

### 2.5 Measurement of cell morphotype ratios in pure *Mc. capsulatus* KN2 cultures during substrate-unlimited batch growth using AI-based analysis

During substrate-unlimited batch growth of the pure *Mc. capsulatus* KN2 culture (Figure 3), time-resolved microscopic analysis was performed in three biological replicates. In total, 277 micrographs were acquired during the experiment, from which the neural network identified 15,124 objects in total (Table 1). Each detected object was characterized using a predefined set of descriptors (Table 3).

**TABLE 3:** List of descriptors assigned to identified objects (i.e., cell morphotypes).

Descriptor	Description	Value
*Object_id*	Unique object identifier containing information on image file, experimental flask number, sampling time, and processing time	format: N_picture_N_flask_Sampling_time_Processing_time_N_object
*Class*	Text-based identifier of the bacterial morphotype class	*monococci*, *diplococci*, *tetracocci*, small_rods, bacilli-like, long_rods, double_cells, arc_cells, thin_rod
*Class_id*	Numeric identifier of the morphotype class	0–*diplococci*, 1–*tetracocci*, 2–*monococci*, 3–long_rods, 4–arc_cells, 5–double_cells, 6–small_rods, 7–thin_rod, 8–bacilli-like
*Area_pixels*	Object area measured in pixels	[pixels]
*Area_μm2*	Calculated object area derived from area_pixels	μm^2^; pixel-to-area conversion was based on a scale bar of 5 μm corresponding to 46 pixels, such that: area_μm^2^ = area_pixels × (5/46)^2^
*Confidence*	Confidence score of object classification, representing the probability assigned by the model to its prediction	range: 0–1

Because the experiment was conducted using a pure (satellite-free) culture of *M. capsulatus* KN2, sporadically detected satellite bacteria (*n* < 10) were excluded from further analysis, as they did not constitute a statistically meaningful contribution.

### 2.6 A cell cycle model for *Methylococcus capsulatus*

By analogy with the eukaryotic cell cycle, the bacterial cell cycle can be conditionally divided into G1/S/G2/M phases (Figure 2). For the formulation of the cell cycle model, the G1/S/G2/M nomenclature was adopted instead of the classical bacterial B/C/D periods, because the D period represents an overlap of the G2 and M phases, during which *monococci* elongate and transition into *diplococci*. To construct a mathematical model of the *Methylococcus capsulatus* cell cycle, the following assumptions were used:

1. the culture is asynchronous and grows in the exponential (log) phase; the specific growth rate (μ) is equal to the maximum specific growth rate (μ = μ_*max*_) and remains constant over time during cultivation (μ_*max*_(t) = const); *monococci* (mono) correspond to cells in the G1/S/G2 phases;

2. *diplococci* (diplo) correspond to cells in the M-phase; *tetracocci* (tetra) correspond to cells in the M-phase and represent a subpopulation of *diplococci*; t_*G1/S/G2*_ denotes the cumulative duration of the G1/S/G2 phases (growth phase), occupied by *monococci* [h];

3. *t_M_* denotes the duration of the M phase (cytokinesis), occupied by *diplococci* and *tetracocci* [h];

4. *f*_*mono*_(*t*) is the fraction of *monococci* in the culture at time *t*;

5. *f*_*diplo*_(*t*) is the fraction of *diplococci* in the culture at time *t*;

6. *f*_*tetra*_(*t*) is the fraction of *tetracocci* in the culture at time *t*.

The maximum specific growth rate μ_*max*_ [1/h] of the culture during substrate-unlimited batch cultivation in the exponential phase was determined experimentally (Figure 3). The corresponding cell doubling time *t_d_* [h], i.e., the generation time, was calculated as:


$$t_{d}=\frac{\ln2}{\mu_{\max}}$$ (2)

From the cell cycle scheme (Figure 2), it follows that:


$$t_{d}=t_{G\,1/S/G\,2}+t_{M}$$ (3)

The fractions of cell morphotypes are defined as:


$$f_{m\,o\,n\,o}+f_{d\,i\,p\,l\,o}+f_{t\,e\,t\,r\,a}=1$$ (4)

$$f_{G\,1/S/G\,2}\,\left(t\right)=f_{m\,o\,n\,o}\,\left(t\right)$$ (5)

$$f_{M}\,\left(t\right)=f_{d\,i\,p\,l\,o}\,\left(t\right)+f_{t\,e\,t\,r\,a}\,\left(t\right)$$ (6)

For an asynchronous population undergoing steady exponential growth, the fraction of cells in the final phase (M-phase, with duration *t_M_*) is described by the following equation (derivation provided in Supplementary Appendix 1):


$$f_{M}\,\left(t\right)=2^{\left[t_{M}\,\left(t\right)\,/\,t_{d}\right]}-1$$ (7)

solving this equation for *t_M_* yields:


$$t_{M}\,\left(t\right)=t_{d}\cdot \ln\left(f_{M}\,\left(t\right)+1\right)$$ (8)

and consequently:


$$t_{G\,1/S/G\,2}\,\left(t\right)=t_{d}-t_{M}\,\left(t\right)$$ (9)

#### 2.6.1 Interpretation of morphotype dynamics

1. An increase in (*f*_*diplo*_ + *f*_*tetra*_) relative to *f*_*mono*_ corresponds to an increase in *t*_*M*_ or, alternatively, a decrease in the doubling time *t*_*d*_ (i.e., an increase in μ). In batch cultures, this may indicate elongation of the M phase due to stress factors (e.g., oxygen or methane limitation, as observed during the stationary phase), or a transition from *lag* to *log* phase characterized by increasing μ.

2. A decrease in *f*_*diplo*_ + *f*_*tetra*_ (i.e., a decrease in *f*_*m*_) relative to *f*_*mono*_ corresponds to shortening of the M phase (accelerated division) or a reduction in μ (increase in *t*_*d*_), which is typical for the transition toward stationary phase, where cells accumulate in early cell cycle stages and the morphotype distribution approaches uniformity (*f*_*tetra*_ ≈ *t_*M*_/t_*d*_* → small values).

3. An increase in the fraction of *tetracocci* relative to *diplococci* may indicate alterations in division plane orientation (e.g., due to stress or temperature effects), thereby affecting the overall M-phase fraction *f*_*M*_.

## 3 Results

### 3.1 Training the neural network to recognize cell morphotypes in a mixed culture based on *Methylococcus capsulatus* KN2

**FIGURE 6:**
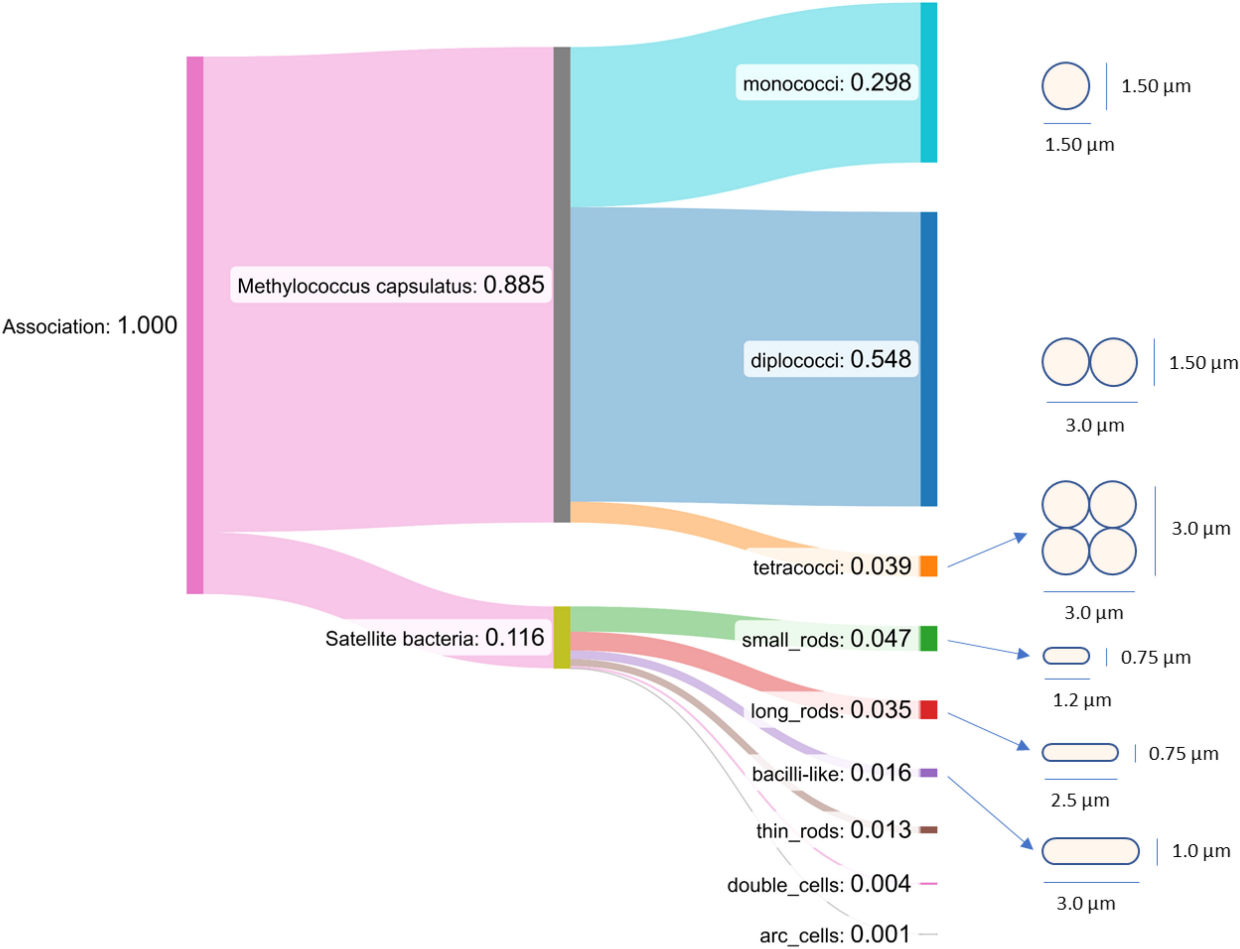
The fractional composition (f_i_) of the active industrial mixed methanotrophic consortium based on *Methylococcus capsulatus* KN2, used for non-sterile cultivation on natural gas methane in a continuous-flow bioreactor, indicating the morphotypes and average cell sizes (Figure 1). The species names of the satellite bacteria are not provided, as this constitutes part of the technological know-how. This fractional composition of the cell morphotypes in the association was obtained during neural network training; a total of 50,410 objects were identified across 250 experimental images from various experiments, collectively using the training and validation datasets (Table 2). The diagram was created on the website https://sankeymatic.com/.

To train and validate the neural network, an active industrial methanotrophic bacterial consortium based on *Methylococcus capsulatus* KN2, cultured in preliminary experiments under nonsterile conditions in bioreactor operated in continuous mode [D = 0.15–0.25 (1/h)] was used, where the consortium contained all morphotypes of producer and satellite bacteria cells (Figures 1, 6 and Table 2). Whereas, the dynamics of the ratio of producer cell morphotypes (*monococci, diplococci, tetracocci*) in a substrate-unlimited batch mode was studied separately using a pure culture of *M. capsulatus* KN2 without satellite bacteria (Table 1).

**FIGURE 7:**
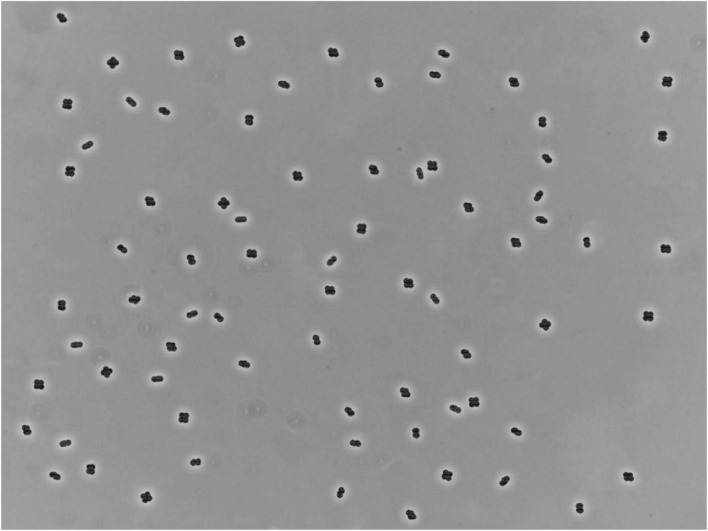
Example of a synthetic image containing various tetracoccus projections.

**FIGURE 8:**
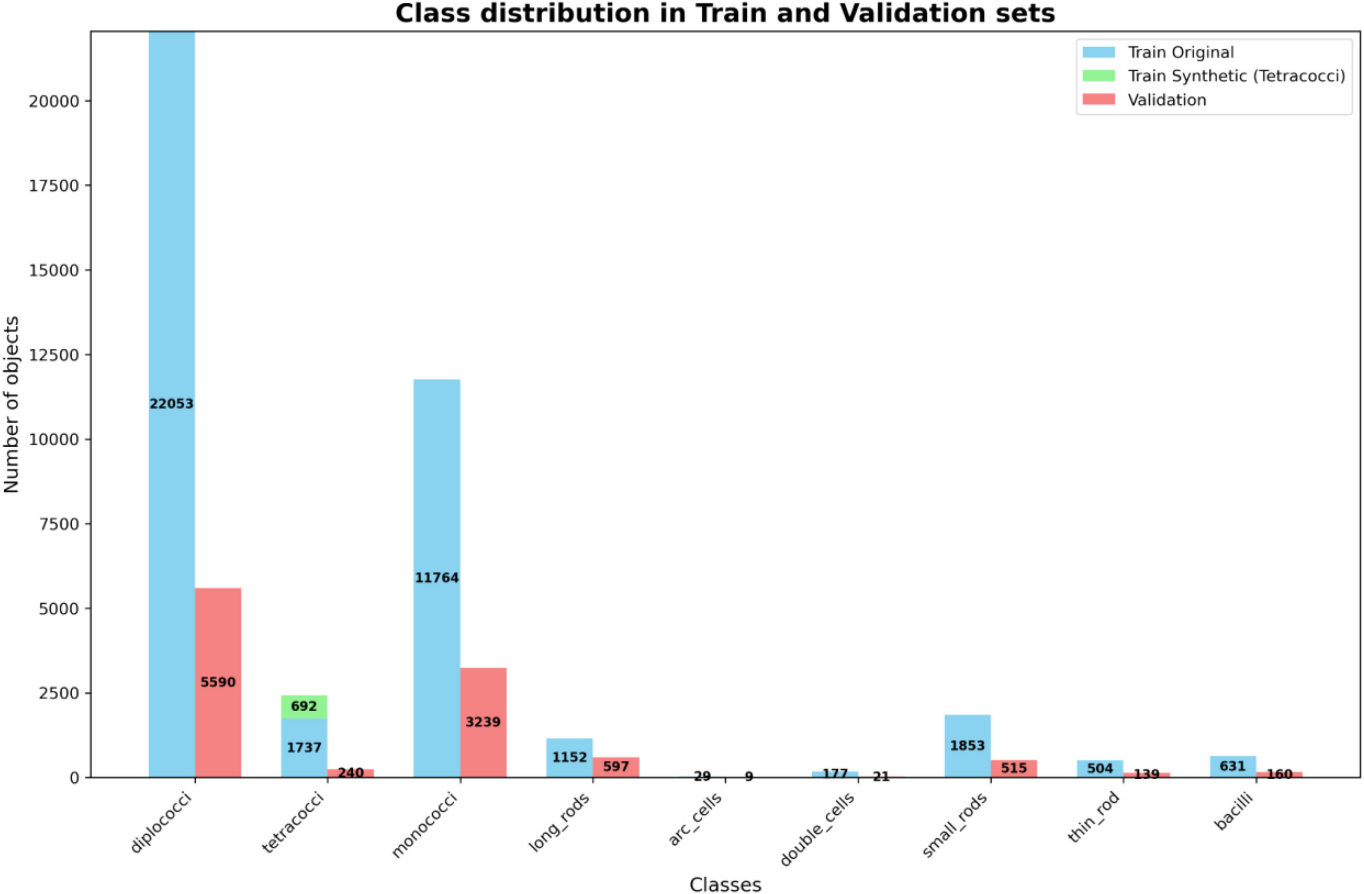
The dataset used for neural network training (Training: *m* = 200 photographs, *n* = 39,900 objects; Validation: *m* = 50 photographs, *n* = 10,510 objects), including synthetic data (*m* = 12 images with *n* = 692 objects), for recognizing cell morphotypes in the active industrial mixed methanotrophic consortium based on *Methylococcus capsulatus* KN2 during non-sterile continuous cultivation in a bioreactor. The original data are provided in Table 2.

The training set of experimental photographs consisted of 250 micrographs of a mixed active industrial culture based on *Methylococcus capsulatus* KN2 (Table 2). All images were manually annotated in CVAT and randomly divided into Training (*m* = 200 micrographs) and Validation (*m* = 50 micrographs) subsets. The *tetracocci* class was underrepresented, so an additional 692 synthetic objects of this morphotype (Figure 5) were generated on 12 synthetic images (Figure 7); the final training set was increased up to 262 micrographs (Figure 8 and Table 2).

Despite augmentation, class imbalance persisted, leading to (i) model overfitting on dominant morphotypes, systematically missing rare classes, (ii) fragmented masks and low confidence in their detection, and (iii) loss of the information content of common metrics (i.e., mAP). To compensate it, (i) a weighted loss function with increased error weighting for rare classes and (ii) two-stage training: pre-training on an artificially balanced subset followed by fine-tuning on the full set has been used.

**FIGURE 9:**
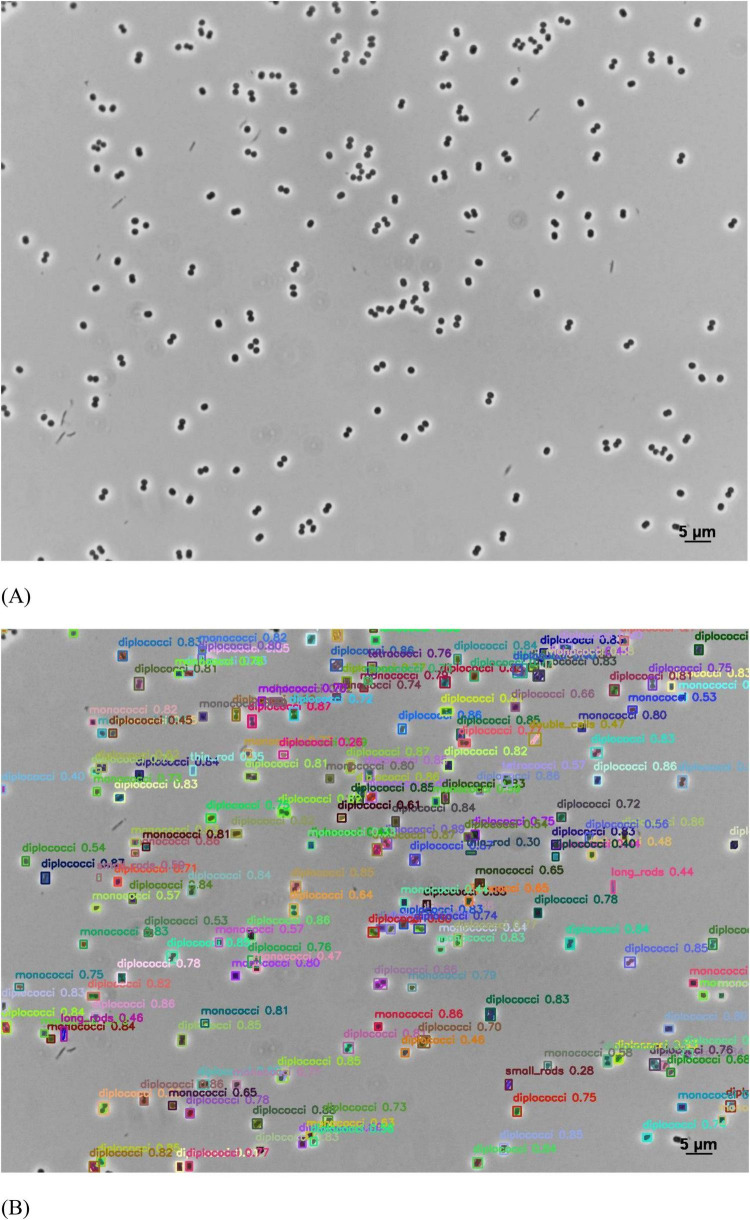
An example of **(A)** the original image and **(B)** the image with object detection and segmentation by the neural network. The micrograph shows an active industrial mixed methanotrophic consortium based on *Methylococcus capsulatus* KN2 during non-sterile continuous cultivation in a bioreactor. Each recognized object is enclosed in a colored bounding box indicating the cell morphotype and the confidence value (identification reliability level). The object area value is not displayed on the photograph. The text color is randomized to facilitate visual perception/analysis of the image. There are examples of close-up views on similar segmented images and some particular objects in Supplementary Figure 1 and Supplementary Table 5.

The trained model demonstrates an average accuracy of 88% for morphotype classification; however, for object detection and instance segmentation tasks, this metric is of auxiliary nature. The primary quality assessment is the mean average precision (mAP). In this case, the mAP@0.5:0.95 metric was used—the average mAP value over Intersection over Union (IoU) thresholds from 0.5 to 0.95 with a step of 0.05. This metric shows how accurately the model predicts object shape. The obtained value of mAP@0.5:0.95 = 0.52 indicates acceptable accuracy of object localization and shape prediction and is sufficient for practical application of the model in the analysis of micrographs of methanotrophic associations. Examples of the neural network’s operation on the original images are shown in Figure 9.

Across the original 250 photographs, 50,410 objects of nine morphotypes were automatically identified, and the proportional composition of the bacterial consortium was determined. It was established that under non-sterile industrial continuous cultivation conditions in bioreactor, the cell of the producer strain constitutes 88.5% and the satellite bacteria 11.5% of the total population (Figure 6 and Table 2). The ratio of producer cell morphotypes (*monococci*, *diplococci*, *tetracocci*) solely for *Methylococcus capsulatus* KN2 within the complex association is: *monococci* + *diplococci* + *tetracocci* = 44,623 objects (Table 2); *monococci*− 0.336, *diplococci* − 0.619, *tetracocci*− 0.044 (for comparison with data obtained from the pure culture of *Methylococcus capsulatus* KN2, see Table 4). Indeed, within the complex association these proportions are reduced due to the presence of satellite bacteria (Figure 6).

**TABLE 4:** Statistical analysis by cell morphotype classes of the pure *Methylococcus capsulatus* KN2 culture (combined dataset from three independent flasks) during substrate-unlimited batch growth with a stationary specific growth rate μ_*max*_ = 0.223 [1/h].

Cultivation time, [h] (*k*)	Morphotype (*i*)	*n*_*k,i*_	*f*_*k,i*_	*S_*k*,i_* (*area_μm2*) av (med) ± *sd* [μm^2^]	$\frac{S_{k,d\,i\,p\,l\,o\,c\,o\,c\,c\,i}}{S_{k,m\,o\,n\,o\,c\,o\,c\,c\,i}}$	Shapiro-Wilk test for *area_μm2*		
						*W*	*P*	*H*_0_
3	*Monococci*	914	0.3542	1.694 (1.796) ± 0.452	1.431	0.9161	0	Not normal
	*Diplococci*	1,574	0.6100	2.424 (2.387) ± 0.547		0.9910	0	Not normal
	*Tetracocci*	92	0.0356 1	3.390 (3.330) ± 0.92		0.9857	0.4149	Normal
	Total	2, 580						
5	*Monococci*	1,124	0.3265	1.757 (1.802) ± 0.397	1.464	0.9468	0	Not normal
	*Diplococci*	2,196	0.6380	2.574 (2.540) ± 0.597		0.9884	0	Not normal
	*Tetracocci*	122	0.0354	3.820 (3.830) ± 0.83		0.9916	0.6766	Normal
	Total	3,442	1					
7	*Monococci*	1,101	0.2727	1.878 (1.914) ± 0.404	1.438	0.9665	0	Not normal
	*Diplococci*	2,772	0.6868	2.701 (2.658) ± 0.589		0.9914	0	Not normal
	*Tetracocci*	163	0.0403 1	3.740 (3.690) ± 0.85		0.9844	0.0636	Doubtful
	Total	4,036						
9	*Monococci*	1, 462	0.2885	1.881 (1.890) ± 0.398	1.462	0.9626	0	Not normal
	*Diplococci*	3,377	0.6666	2.750 (2.717) ± 0.604		0.9926	0	Not normal
	*Tetracocci*	227	0.0448 1	3.860 (3.830) ± 0.80		0.9904	0.1399	Normal
	Total	5,066						

*n*, number of cells in the combined dataset from three independent flasks; *f*, fraction of cells in the dataset; *area_μm2*, calculated “shadow “ area of the cell; *av*, average; *med*, median; *sd*, standard deviation; *W*, value of Shapiro-Wilk normality test; *P*, *P*-value; H_0_, null-hypothesis: the normality of the dataset is not significant if *P* < 0.05.

**FIGURE 10:**
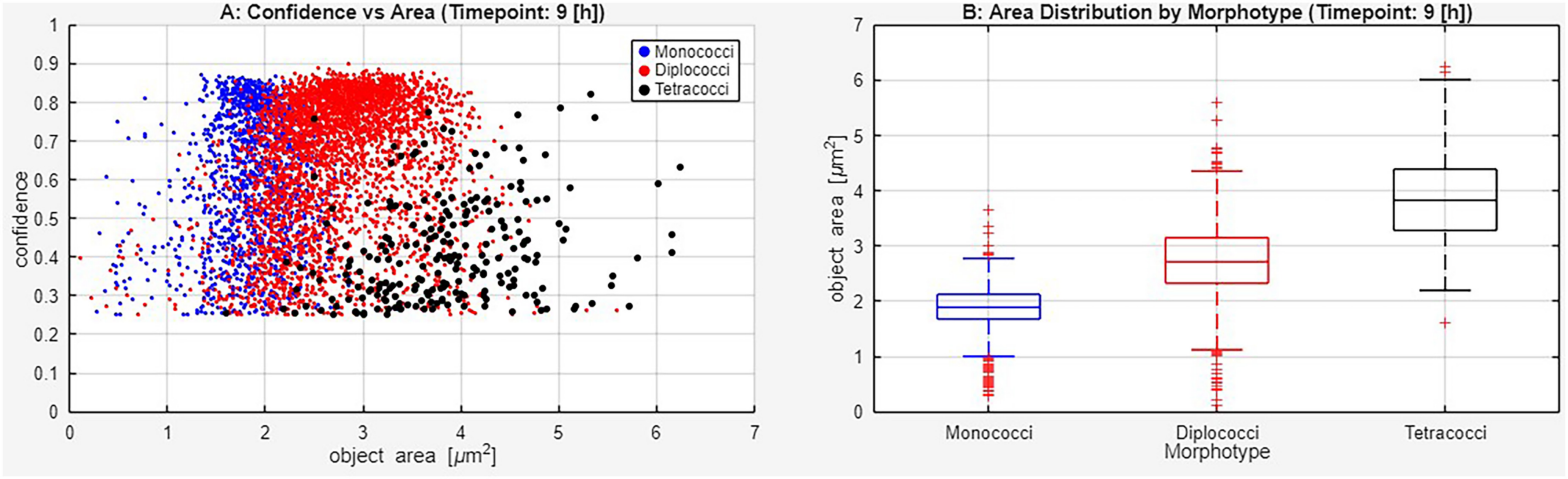
Example of the analysis of **(A)** the distribution of experimental data (confidence vs. object area) and **(B)** box plots by cell morphotype classes at *t* = 9 [h] for the pure *Methylococcus capsulatus* KN2 culture during substrate-unlimited batch growth with a stationary specific growth rate ì_*max*_ = 0.223 [1/h]. Analysis for the remaining time points (*t* = 3, 5, 7 [h]) is not presented here to reduce space.

**FIGURE 11:**
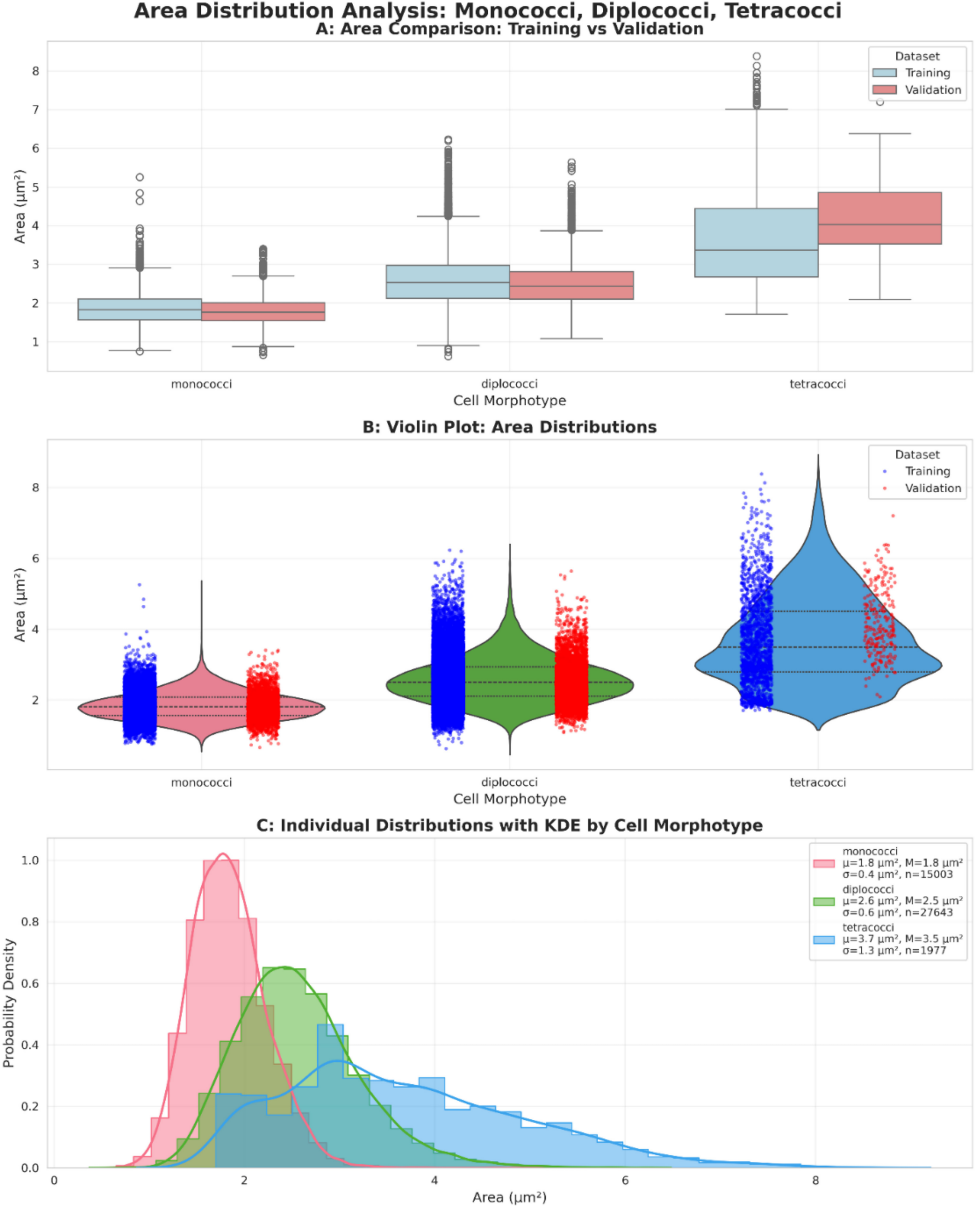
Analysis of the distribution of “shadow” areas for the cell morphotypes of the producer *Methylococcus capsulatus* KN2 (i.e., *monococci*, *diplococci*, *tetracocci*) cultivated under non-sterile conditions in continuous mode in bioreactor used during neural network training. **(A)** Box plot and **(B)** violin plot of the “shadow” area distributions for cell morphotypes separately for the Training and Validation datasets; **(C)** probability density distribution of “shadow” areas for cell morphotypes in the combined dataset (Training and Validation). Numerical data are presented in Table 2.

For each detected object (i.e., cell morphotype), following quantitative parameters were calculated: (i) identification confidence—the reliability level of automatic identification (for example, Figure 10); and (ii) the area of the cell’s projected “shadow” [μm^2^] (Tables 2–4). The “shadow” area was used as a quantitative feature in subsequent statistical analysis (Supplementary Tables 3, 4, Table 4, and Figures 10, 11).

#### 3.1.1 Statistical tests of results obtained during neural network training on the complex industrial consortium based on the producer *Methylococcus capsulatus* KN2

The experimental data used for neural network training were obtained from an active industrial complex methanotrophic consortium based on *Methylococcus capsulatus* KN2 under non-sterile continuous cultivation in a bioreactor (Table 2 and Figures 8, 9) and were analyzed using statistical tests according to the following scheme:

1. Normality test using the Shapiro-Wilk, D’Agostino’s χ^2^, and Kolmogorov-Smirnov tests for all data arrays of “shadow” areas, grouped by morphotype and dataset;

2. Pairwise comparison of the distribution densities of “shadow” areas for corresponding cell morphotypes from the Training vs. Validation datasets using the non-parametric Mann-Whitney U test;

3. Merging objects from the Training and Validation datasets into a single combined dataset;

4. Re-testing the normality of the combined dataset using the Shapiro-Wilk, D’Agostino’s χ^2^, and Kolmogorov-Smirnov tests;

5. Assessing differences between the distribution densities of “shadow” areas across cell morphotypes using the non-parametric Kruskal-Wallis H test;

6. Pairwise comparison of the distribution densities of “shadow” areas for cell morphotypes using the non-parametric Mann-Whitney U test;

Normality analysis of the “shadow” area distributions for the corresponding cell morphotypes, grouped into Training and Validation datasets (Figures 11B,C) using the Shapiro-Wilk, D’Agostino’s χ^2^, and Kolmogorov-Smirnov tests, revealed a non-normal probability density distribution (*p* < 0.001) within all cell morphotypes in each dataset (Training and Validation). This fact necessitated the use of non-parametric statistical methods.

The non-parametric Mann-Whitney U test for pairwise comparison of “shadow” area distributions for the corresponding producer cell morphotypes of *Methylococcus capsulatus* KN2 (i.e., *monococci, diplococci, tetracocci*) within the mixed industrial consortium between the Training and Validation datasets (Figures 11A,B) revealed no differences (*p* < 0.001) in the distributions of corresponding morphotypes. Therefore, the identified objects from both datasets were merged into a single unified dataset for further statistical analysis (e.g., Figure 11C) and re-tested for normality of the “shadow” area distributions for the corresponding cell morphotypes using the Shapiro-Wilk, D’Agostino’s χ^2^, and Kolmogorov-Smirnov tests. These tests again confirmed a non-normal probability density distribution (*p* < 0.001) within all morphotypes (Supplementary Table 3 and Figure 11C). All distributions are characterized by positive skewness (right-skewed) and are leptokurtic (peaked), except for *tetracocci*, which are mesokurtic (Supplementary Table 3).

Comparison of the “shadow” area distributions for the three cell morphotypes of *Methylococcus capsulatus* KN2 (Figure 11A) using the non-parametric Kruskal-Wallis H test revealed statistically significant differences between the morphotypes (H-statistic: 15500.6372; *p*-value: 0.0000). Subsequent pairwise comparisons using the Mann-Whitney U test with Bonferroni correction (Supplementary Table 4) also showed statistically significant differences (*p* < 0.001). The effect sizes (r) in all comparisons were large:

1. Greatest difference between *tetracocci* and *monococci* (*r* = 0.897);

2. Large difference between *monococci* and *diplococci*;

3. Smallest, yet still large, difference between *diplococci* and *tetracocci* (*r* = 0.558).

### 3.2 Dynamics of biomass growth of the pure culture *Methylococcus capsulatus* KN2 in a substrate-unlimited batch experiment

Experimental measurements of the dynamics of cell morphotype ratios during substrate-unlimited growth were performed on a pure culture of *Methylococcus capsulatus* KN2 (i.e., without satellite bacteria). Exponential growth (Equation 1) of the pure *Methylococcus capsulatus* KN2 culture with μ_*max*_ = 0.223 [1/h] began after a *lag*-time = 1.50 [h] post-inoculation (during which *lag*-time μ_*max*_ = 0 [1/h]) (Figure 3). Since *OD*_600_ in the experiment did not exceed 1 [OU], it can be stated that sample analysis was conducted during the early exponential growth phase of the culture. Thus, the maximum specific growth rate (μ_*max*_) during the substrate-unlimited batch growth remained unchanged (i.e., was stationary) throughout the entire cultivation period (Figure 3). According to Equation (2), the cell doubling time (generation time) *t_d_* = 3.11 [h].

**FIGURE 12:**
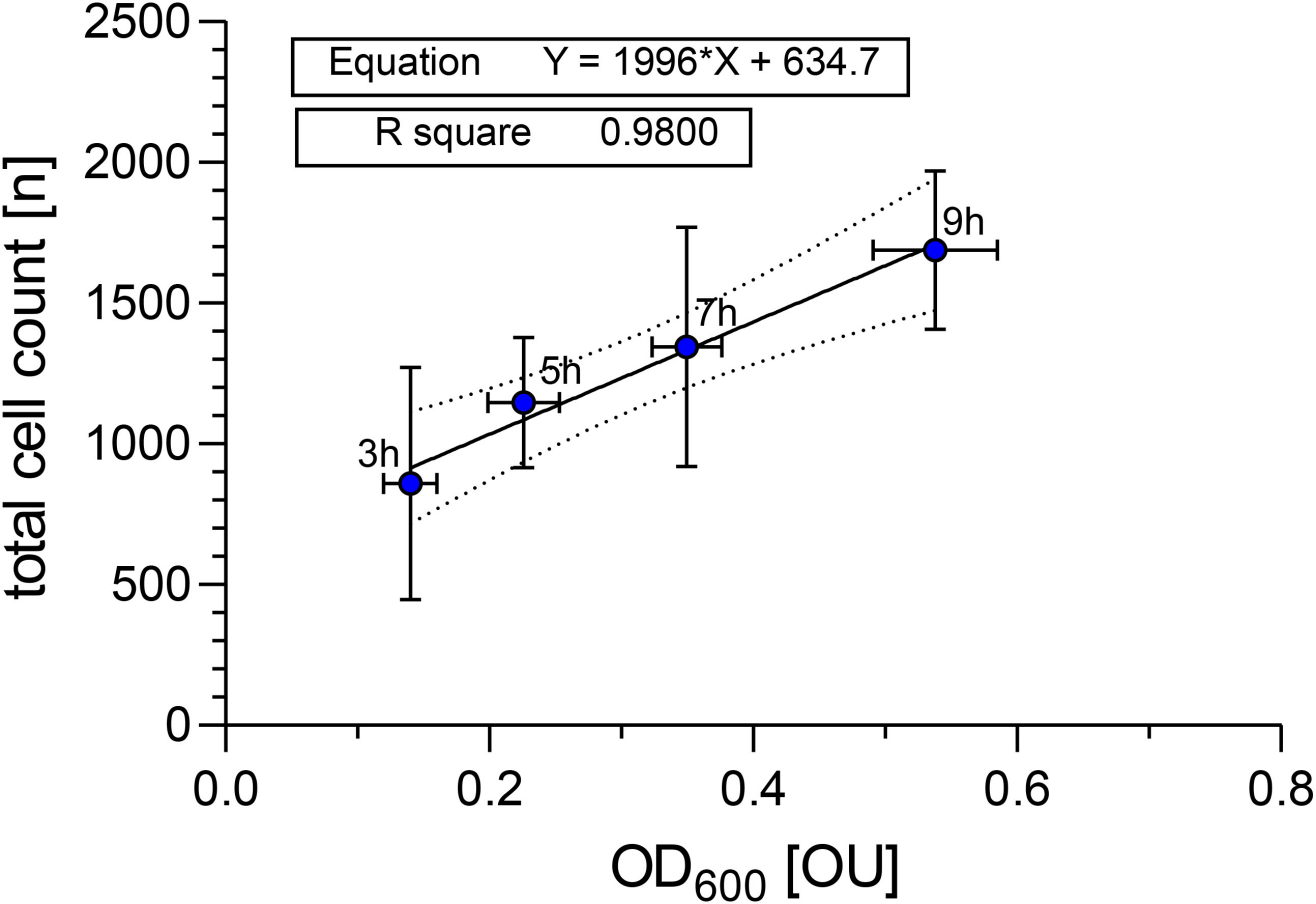
Dependence of the total number of cells morphotypes *n* identified by the neural network (Table 1) on the measured *OD*_600_ (Figure 3) averaged for three biological replicates (*average* ± *sd*, *n* = 3). Dotted lines—95% confidence bands of the best-fit line.

A statistically significant correlation was observed between the experimentally measured *OD*_600_(*t*) (Figure 12) and the total number of cells *n*(*t*) identified by the neural network (Table 1) across *m* analyzed micrographs collected during the substrate-unlimited batch growth of the pure *Methylococcus capsulatus* KN2 culture: Pearson *r* = 0.9899, *R*^2^ = 0.9800, *p* = 0.0101. This allowed for regression analysis (Figure 12) and the identification of a linear relationship *n*(*OD*_600_) c *R*^2^ = 0.9800. However, in this case, the number of identified cells in the dataset cannot be linked to the volume of the liquid phase they occupied, making it impossible to relate *OD*_600_ to cell concentration. Consequently, the identified relationship cannot serve as a predictive tool. To overcome this limitation, it is necessary to use a flow-through microscopy chamber with a fixed volume in the microscope’s field of view.

### 3.3 Analysis of experimental data on the dynamics of morphotype content and cell “shadow” areas of a pure *Methylococcus capsulatus* KN2 culture during substrate-unlimited batch growth using AI technologies

As a result of the substrate-unlimited batch cultivation of the pure *Methylococcus capsulatus* KN2 culture across three biological replicates and four time points, 277 experimental images were acquired, on which 15,124 objects were automatically identified (Table 1). Since this experiment was conducted on a pure culture of *Methylococcus capsulatus* KN2, only the morphotypes *monococci*, *diplococci*, *tetracocci* were used for analysis. Objects classified as “satellites” were identified only in trace amounts (*n* < 10 per time point in each replicate) and were therefore excluded from the analysis due to low statistical significance.

When analyzing images from a microscope, the AI algorithm operates with the 2D projection of a cell onto a plane, i.e., the cell’s “shadow.” The AI algorithm identifies an object, classifies the cell morphotype, determines the object’s edge, and calculates the area of the object’s “shadow” in [pixels], which is then converted to [μm^2^]. For each identified object (i.e., *monococci, diplococci, tetracocci*) in the experimental dataset, quantitative characteristics were calculated: *area_μm2*—the calculated “shadow” area of the object [μm^2^]; *confidence*—the reliability level (probability) of recognition assigned by the model to its prediction for the identified object (Table 3). The *area_μm2* data arrays from independent biological replicates (independent flasks) at each time point for each cell morphotype class were tested for normality using the Shapiro-Wilk test. Since the probability density distribution of *area_μm2* was found to be non-normal (*p* < 0.001), the arrays were compared using the non-parametric Kruskal-Wallis test for multiple comparisons. No differences were detected between them (*p* < 0.001). Therefore, the replicates from independent flasks at each time point were pooled for data quality assessment and analysis of the dynamics of cellular culture parameters (e.g., Figure 10).

Analysis of the distribution of bacterial cell areas in the dataset pooled across time points for the three morphological types (*monococci, diplococci, tetracocci*) over the 9-h experiment revealed the following patterns:

1. Heterogeneity in normality of distributions (Shapiro-Wilk test, Table 4) across morphological classes:

a. *Diplococci* and *monococci* show a statistically significant deviation from a normal distribution (*p* < 0.001) at all-time points;
b. *Tetracocci* maintain a normal distribution at most time points (3, 5, 9 h), except for the 7-h point where the distribution becomes questionable;

2. Pronounced temporal dynamics of the fractional composition and “shadow” areas of cell morphotypes (Tables 4, 5 and Figure 4):

a. The Kruskal-Wallis test revealed highly significant differences between time groups for all classes:

i. *diplococci*: H = 368.61, *p* < 0.001;
ii. *monococci*: H = 148.21, *p* < 0.001;
iii. *tetracocci*: H = 18.11, *p* = 0.0004;

3. Inter-class differences:

a. Combined analysis of all data showed extremely significant differences between the classes (*H* = 6502.35, *p* < 0.001), indicating fundamental morphological distinctions among the three types of cocci.

The non-normality of the “shadow” area distributions for *diplococci* and *monococci* may indicate:

- Population heterogeneity—the presence of subpopulations with different sizes;
- Asynchronous division—cells are at different stages of the cell cycle;
- Environmental influence—uneven exposure to cultivation conditions;
- Presence of dividing cells—inclusion of cells in the process of division in the analysis;

In contrast, the normal distribution of “shadow” areas for *tetracocci* suggests:

- Population homogeneity—more synchronized division and growth;
- Morphological stability—lower size variability;
- Environmental robustness—a more stable response to changes in conditions;

*Diplococci* and *monococci* demonstrate a statistically significant deviation from a normal distribution at all cultivation time points, which is likely associated with asynchrony in cell division and the presence of subpopulations. These features must be taken into account when planning further experiments and during the statistical analysis of the data.

#### 3.3.1 Dynamics of the fractional composition of cell morphotypes in the pure *Methylococcus capsulatus* KN2 culture during substrate-unlimited batch growth

Fractional sum of cell morphotypes in pure culture is equal to 1 (Equation 4). During the substrate-unlimited batch experiment, a statistically significant dynamic in the fractional composition of the pure *Methylococcus capsulatus* KN2 culture is observed (Figure 4A and Table 4). The most pronounced dynamic changes during cultivation were demonstrated by *monococci* and *diplococci*. From the start of the cultivation, the fraction of *diplococci* increased almost linearly from 0.61 [3(h)] to 0.68 [7(h)] and then stabilized at 9[h]. Concurrently, the fraction of *monococci* decreased almost linearly from 0.35 [3(h)] to 0.27 [7(h)] and then stabilized at 9[h]. The changes in the proportions of *monococci* and *diplococci* demonstrate a statistically significant negative correlation with each other—if the fraction of *diplococci* increases, the fraction of *monococci* decreases correspondingly, and *vice versa* (Pearson *r* = −0.996, *p* = 0.004). In contrast, the fraction of *tetracocci* varies only slightly [from 0.035 at 3(h) to 0.044 at 9(h)] and does not correlate with the fractional content of *monococci* (Pearson *r* = −0.631, *p* = 0.369) or *diplococci* (Pearson *r* = 0.564, *p* = 0.436). Based on these findings, it can be concluded that a strict relationship exists between the fractional contents of *monococci* and *diplococci* in the culture. Meanwhile, the fractional content of *tetracocci* appears to be random, as it is not statistically linked to the fractional content of *diplococci*. Although, in this research, the morphotype *tetracocci* is considered to be a sub-population of the *diplococci* morphotype.

The dynamic change in the ratio of the fractional composition of cell morphotypes (*monococci, diplococci, tetracocci*) in the pure culture of *Methylococcus capsulatus* KN2 during the substrate-unlimited batch experiment (Table 4 and Figure 4A) indicates a change in the duration of cell cycle phases during cultivation.

#### 3.3.2 Dynamics of “shadow” areas of cell morphotypes in the pure *Methylococcus capsulatus* KN2 culture during substrate-unlimited batch growth

During the 9-h substrate-unlimited batch cultivation of the pure *Methylococcus capsulatus* KN2 culture, an almost linear increase in the mean “shadow” area for all cell morphotypes was observed (Figure 4B and Table 4): *monococci*—from 1.69 to 1.88 [μm^2^] (+11.2%); *diplococci*—from 2.42 to 2.75 [μm^2^] (+13.6%); *tetracocci*—from 3.39 to 3.86 [μm^2^] (+13.8%). This indicates an increase in the “critical mass” of the cell (Figure 2) during exponential growth, i.e., the cell mass and volume at which the cells initiate the S-phase (i.e., C-period) of the cell cycle and proceed to division.

As a first approximation, *monococci* are perfect spheres. Therefore, from the experimentally measured “shadow” area (S-area_μm2, Table 4), it is possible to calculate (Supplementary Appendix 2) the cell radius (r), intracellular volume (V), periplasmic surface area of the cell (A), and the surface-to-volume ratio (A/V) (Table 5). The cell radius (r) is the only variable that influences the size of the geometric shape and, consequently, the “shadow” area of *monococci*.

**TABLE 5:** Changes in the geometric parameters of *monococci* and *diplococci* during substrate-unlimited batch cultivation.

Time [h]	*Monococci* (direct calculations based on the mean “shadow” area, Supplementary material 2)						
	n*	f*	area_μm2* ± *sd* [μm^2^]	*r* [μm]	*V* [μm^3^]	*A* [μm^2^]	*A/V* [μm^2^/μm^3^]
3	914	0.3542	1.694 ± 0.452	0.734	1.659	6.776	4.085
5	1,124	0.3265	1.757 ± 0.397	0.748	1.752	7.028	4.012
7	1,101	0.2727	1.878 ± 0.404	0.773	1.936	7.512	3.880
9	1,462	0.2885	1.881 ± 0.398	0.773	1.941	7.524	3.877
Diplococci (calculations are based on certain assumptions, Supplement 2)							
3	1,574	0.6100	2.424 ± 0.547	0.602	1.716	7.735	4.507
5	2,196	0.6380	2.574 ± 0.597	0.613	1.855	8.318	4.483
7	2,772	0.6868	2.701 ± 0.589	0.634	2.013	8.635	4.290
9	3,377	0.6666	2.750 ± 0.604	0.634	2.050	8.837	4.326

where: *, empirical parameters; *n*, number of cells in the combined dataset; *f*, fraction within the culture; *area_μm2*, calculated “shadow” area; *r*, cell radius; *V*, intracellular volume; *A*, periplasmic surface area; *A/V*, surface-to-volume ratio; *sd*, standard deviation.

Thus, the geometric parameters of *monococci* changed during substrate-unlimited batch cultivation are varying: the mean cell radius increased from 0.734 to 0.774 [μm] (+5.4%); intracellular volume from 1.659 to 1.941 [μm^3^] (+16.9%); surface area from 6.776 to 7.524 [μm^2^] (+11.03%); and the surface-to-volume ratio decreased from 4.085 to 3.877 [μm^2^/μm^3^] (−5.1%) (Table 5). An increase in the cell radius of *monococci* leads to an increase in intracellular volume and surface area, but at the same time reduces the *A/V* ratio.

The analysis of “shadows” from *diplococci* is more complex (Supplementary Appendix 2) because the number of variables involved in forming the geometric shape and influencing the “shadow” area increases compared to *monococci*: *r*–cell radius; *h*–overlap depth of the spheres; φ–the YZ tilt angle of the object relative to the projection plane. In the case of *tetracocci*, an additional variable is added (in addition to those for *diplococci*): ϕ–the XZ tilt angle of *tetracocci* relative to the projection plane. Thus, two identical *diplococci* or *tetracocci* objects, oriented at different angles to the projection plane (i.e., φ or/and ϕ), can produce significantly different “shadow” areas on that plane. This explains the width of the probability density distribution peak for the experimentally measured “shadow” areas of *Methylococcus capsulatus* KN2 cell morphotypes (Figure 11C): the sharpest and highest peak belongs to *monococci*, where the peak width is determined solely by the natural variation in cell radius (variability only in *r*); the distribution peak for *diplococci* is lower and broader (variability in *r, h, φ*); and the lowest and broadest distribution peak is for *tetracocci* (variability in *r, h, φ, ϕ*).

Based on the adopted assumptions, the average geometric dimensions of *diplococci* were calculated (Supplementary Appendix 2). The average geometric parameters of *diplococci* changed during substrate-unlimited batch cultivation: the mean cell radius increased from 0.602 to 0.634 [μm] (+5.3%); intracellular volume from 1.716 to 2.050 [μm^3^] (+19.5%); surface area from 7.735 to 8.837 [μm^2^] (+14.2%); and the surface-to-volume ratio decreased from 4.507 to 4.326 [μm^2^/μm^3^] (−4.1%) (Table 5). Overall, the cell radius of *diplococci* is smaller than the cell radius of *monococci*, which is expected since new *monococci* cells grow during the G1 phase. The increase in cell radius of *diplococci* during cultivation leads to an increase in intracellular volume and surface area, while simultaneously reducing the *A/V* ratio. It is important to note that the average cell radius of *diplococci* is smaller than that of *monococci*.

It is noteworthy that, on average, the ratio of “shadow” areas *S_*diplococci*_/S_*monococci*_* = 1.45 ± 0.02 across all measured time points (Table 4). The constancy of this ratio indicates an interdependence between *S*_*diplococci*_ and *S*_*monococci*_. Furthermore, from the estimation of the intracellular volume of *diplococci*, it follows that it is very close to the intracellular volume of *monococci* (Table 5). This fact is consistent with the understanding that during the cell division cycle, once the *monococci* morphotype reaches a “critical volume/mass” in the G1 phase (B-period), the cell then proceeds to the S/G2 phase (C/D-period) and attains the *diplococci* morphotype through the redistribution of the existing cell volume, rather than by creating new or additional cell volume (Figure 2). After the M-phase, *diplococci* normally separate into smaller *monococci*, which continue to grow in the G1 phase.

In *Methylococcus capsulatus*, the cytoplasmic membrane forms folds, which increases the surface area available for accommodating membrane-bound methane monooxygenase (mMMO). As a result, the total area of the cytoplasmic membrane exceeds that of the periplasmic membrane (*A*_*i*_). Additionally, another form of the enzyme, periplasmic methane monooxygenase (pMMO), is localized in the periplasm.

The increase in the average cell size of *M. capsulatus* during cultivation (Table 5) can be interpreted as an adaptation aimed at enhancing methane utilization efficiency through an increase in the total area of the cytoplasmic membrane and the periplasmic space, which harbor both forms of methane monooxygenases.

#### 3.3.3 Dynamics of cell cycle parameters of the pure *Methylococcus capsulatus* KN2 culture

Experimental data showed that the pure *Methylococcus capsulatus* KN2 culture grew with stationary μ_*max*_ = 0.223 [1/h] during the 9-h substrate-unlimited batch cultivation. According to Equation (2), the biomass doubling time was *t*_*d*_ = 3.11 [h] and remained unchanged throughout the experiment. The biomass doubling time (*t*_*d*_)–represents the duration of the entire cell cycle, which consists of four phases [Equation (3); Figure 2]. *Monococci* within the population represent cells in the G1/S/G2 phase of the cell cycle (Equation 5), while *diplococci* together with *tetracocci* represent cells in the M-phase (Equation 6). A statistically significant negative correlation (Pearson *r* = −0.996, *p* = 0.004) was identified between the fractional content of *monococci* (*f*_*monococci*_) and *diplococci* (*f*_*diplococci*_) in the pure *Methylococcus capsulatus* KN2 culture (Figure 4A). In the cell cycle of *Methylococcus capsulatus* (Figure 2), *tetracocci* are considered a stochastic sub-population of *diplococci* because their fractional content (*f*_*tetracocci*_) does not correlate with either *f*_*monococci*_ or *f*_*diplococci*_. This circumstance does not require the introduction of an additional second-division phase (*diplococci* → *tetracocci*); therefore, for analyzing the duration of cell-cycle phases, cells in the M-phase (i.e., *diplococci*, *tetracocci*) were combined (Equation 6). The duration of the M-phase (*t*_*M*_) relative to the biomass doubling time (*t*_*d*_) during stationary exponential growth with an unchanged maximum specific growth rate (*μ_*max*_*) determines the fraction of *diplococci* and *tetracocci* in the culture population (Equation 7). Thus, if *t*_*d*_, *f*_*diplococci*_, *f*_*tetracocci*_ are known experimentally, it is possible to calculate the duration of the M-phase of the cell cycle (*t_M_*, Equation 8) and, accordingly, the duration of the G1/S/G2-phase of the cell cycle (*t*_*G1/SG2*_, Equation 9).

**FIGURE 13:**
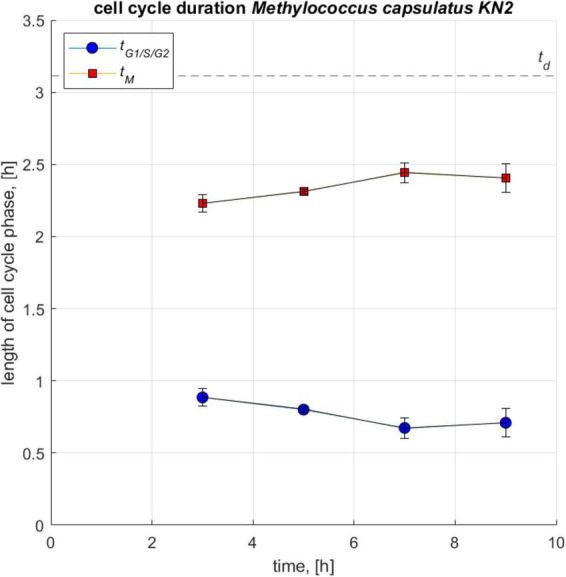
Duration of cell cycle phases of the pure *Methylococcus capsulatus* KN2 culture, calculated based on the experimentally measured μ_*max*_ = 0.223 [1/h] [i.e., t_*d*_ = 3.11 (h)] (Figure 2) and the fractions of cell morphotypes *f*_*mono*_(*t*), *f*_*diplo*_(*t*), *f*_*tetra*_(*t*) (Table 4 and Figure 4A). t_d_—doubling time [h] (Equation 2); t_G1/S/G2_—duration of the combined G1/S/G2-phase containing *monococci* (Equation 9]; *t_M—_*duration of the M-phase containing *diplococci* and *tetracocci* (Equation 8).

Figure 13 presents the calculated durations of the M-phase (*t*_*M*_) and, correspondingly, the G1/S/G2-phase (*t*_*G1/SG2*_) during the 9-h substrate-unlimited batch cultivation of the pure *Methylococcus capsulatus* KN2 culture. As shown in Figure 13, the duration of the M-phase (*t_M_*) y increased from 2.23 [h] @ 3[h] to 2.44 [h] @ 7[h] and then stabilized at 2.40 [h] @ 9 [h]. The accumulation of *diplococci* in the batch culture (Figure 4A) is consistent with the concept of slowed cytokinesis (i.e., a delayed transition from the M-phase to the G1-phase); thus, the increase in *t_M_* indicates elongation of the M-phase, which may be associated with slowed cell division. Given the inverse correlation between the contents of *monococci* and *diplococci*, and assuming the biomass doubling rate (*t*_*d*_) remained constant throughout the experiment, it follows that the calculated growth rate of *monococci* increases, meaning *t*_*G1/S/G2*_ decreases (Figure 13). Most likely, by the 9th h of growth, the *Methylococcus capsulatus* KN2 culture entered a steady-state exponential growth phase, at which point the *monococci*/*diplococci* ratio is expected to remain constant over time, provided μ_*max*_ stays unchanged. The slowing of the M-phase could be due to limitations in the cell’s energy balance resulting from the onset of CH_4_ or O_2_ limitation in the gas phase of the experimental flasks. On average, during the substrate-unlimited batch growth of the pure *Methylococcus capsulatus* KN2 culture with μ_*max*_ = 0.223 [1/h], the M-phase accounted for 76% of the cycle, while the combined G1/S/G2-phase accounted for 24% of *t*_*d*_ = 3.11 [h].

## 4 Discussion

The studied complex active industrial methanotrophic consortium consists of cells of the producer strain—*Methylococcus capsulatus* KN2 (morphotypes: *monococci*, *diplococci*, *tetracocci*) and “satellite” cells (morphotypes: small_rods, bacilli-like, long_rods, double_cells, arc_cells, thin_rod) (Figures 1, 6). In the industrial culture, the “satellite” bacteria perform a stabilizing function for the producer strain, as they are heterotrophs and utilize the metabolic by-products (i.e., exometabolites) of the producer strain, thereby preventing the accumulation of terminal exometabolites that inhibit the growth of the producer strain (Esembaeva et al., 2025). The growth of the industrial producer strain in consortium with “satellites” stabilizes the culture growth and enables cultivation under non-sterile conditions with a high biomass concentration.

For automation and increased accuracy in the microscopic study of the fractional composition of cell morphotypes in the culture, a neural network was applied. The obtained results demonstrate the effectiveness of the proposed methodology based on artificial intelligence technologies for analyzing the population dynamics of the methanotrophic bacterial consortium based on *Methylococcus capsulatus* KN2. The trained convolutional neural network YOLO11x-seg successfully automatically identified 50,410 objects of nine morphotypes across 250 micrographs (in the Training and Validation datasets), enabling the quantitative assessment of the association’s fractional composition (producer: 88.5%, satellites: 11.5% of the total population) under non-sterile continuous cultivation conditions (Figure 6 and Table 2). These data are consistent with previously described proportions in industrial methanotrophic communities, where the dominance of the producer *Methylococcus capsulatus* (90–95%) ensures process stability, and heterotrophic satellites (5–10%) prevent the accumulation of inhibitory exometabolites (Zhuang et al., 2024; Bothe et al., 2002). The role of satellites in utilizing exometabolites, such as organic acids and alcohols, confirms their contribution to maintaining high productivity (*Q_X_*) and biomass concentration (*C_X_*), which is critical for non-sterile continuous SCP production (Zhuang et al., 2024).

The cell cycle of a *Methylococcus capsulatus* culture growing with a constant specific growth rate (μ_*max*_) in the exponential phase (i.e. log-phase) is presented in Figure 3. Analysis of the fractional content of producer cell morphotypes (*monococci*, *diplococci*, *tetracocci*) revealed their connection with the cell cycle phases. According to the model (Figure 2), *monococci* correspond to the combined G1/S/G2 phase, where cells accumulate resources to initiate DNA replication, while *diplococci* and *tetracocci* reflect the M-phase, characterized by septum formation and cytokinesis (Wang and Levin, 2009). In the G2 phase, cells elongate and a bridge (septum) form; however, in bacteria it is difficult to clearly distinguish the end of the G2-phase and the beginning of the M-phase after elongation, the cell begins to divide symmetrically in a single plane. In this context, it is valid to speak of a visual overlap between the G2- and M-phases, which in the bacterial cell cycle is referred to as the D-period (Figure 2). In the M-phase, *diplococci* exist as an encapsulated pair, united within a single polysaccharide capsule that protects the cells from stressful conditions (e.g., low pO2, mechanical stress etc.), making them resilient to environmental conditions (Nakamoto et al., 2023). The presence of the polysaccharide capsule delays the separation of diplo- and *tetracocci* cells during cytokinesis; therefore, the duration of the M-phase is expected to dominate (76% of the time) in the cell cycle of a *Methylococcus capsulatus* culture growing at a steady rate. Thus, the observed longer M-phase (compared to G1/S/G2) in the experiment reflects the natural biology of *Methylococcus capsulatus*. In the substrate-unlimited batch experiment, the proportion of *tetracocci* remained virtually unchanged (Figure 4 and Table 4). The lack of correlation between the fraction of *tetracocci* and other morphotypes (Pearson *r* = −0.631 for *monococci* and *r* = 0.564 for *diplococci*, *p* > 0.3) allows *tetracocci* to be considered as a stochastic subpopulation of *diplococci*, possibly arising from delayed cell separation within the polysaccharide capsule and a transition to the next division cycle in a different plane (Ward et al., 2004). This is consistent with observations on the morphological plasticity of Type I methanotrophs, where capsular structures enhance resistance to oxidative stress and low oxygen levels (Bowman, 2016).

The trained neural network was used to analyze the dynamics of the fractional composition of the pure *Methylococcus capsulatus* KN2 culture—three biological replicates, four time points (3, 5, 7, 9 [h]) (Figure 3), a total of 277 images on which 15,124 cell morphotype objects (*monococci*, *diplococci*, *tetracocci*) were identified (Table 4). In the substrate-unlimited batch experiment with the pure KN2 culture, a significant negative correlation was observed between the fractions of *monococci* and *diplococci* (Pearson *r* = −0.996, *p* = 0.004), with an increase in the fraction of *diplococci* from 0.61 to 0.68 during the first 7 [h] of cultivation. This indicates an elongation of the M-phase (from 2.23 to 2.44 [h]), accounting on average for 76% of the doubling time [*t*_*d*_ = 3.11 (h) at μ_*max*_ = 0.223 (1/h)], while the G1/S/G2-phase decreased to 24%. Such changes may be caused by increasing limitation of substrates (CH_4_ or O_2_) or the accumulation of terminal exometabolites in the batch flasks, leading to slowed cytokinesis and accumulation of cells in the M-phase (Kalyuzhnaya et al., 2013). Similar patterns of asynchronous division have been described for other gammaproteobacteria, where energy deficiency inhibits septum formation (Foster and Davis, 1966). Stabilization of the composition after 7 [h] suggests entry into a steady-state exponential phase, where the morphotype ratio reflects a balance between growth and division.

For a complex culture (i.e., consortium) growing under steady-state conditions on natural gas (∼95% methane, ∼5% C2 + aliphatic homologues), a change in the *monococci:diplococci* (+*tetracocci*) ratio indicates a shift in the duration of cell cycle phases. This possibly signals an impending trend in biomass growth rate alteration due to nutrient limitation, stress, or accumulation of terminal exometabolites. An increase in the proportion of satellites may point to suboptimal cultivation conditions, leading to an excess of substrate for the growth of heterotrophic satellites. For example, this could be dead producer biomass (it is important to identify the source of the problem) or excessive accumulation of terminal exometabolites from the producer’s metabolism—alcohols (e.g., methanol), including those from the oxidation of C2 + aliphatic homologues due to an increased share of C2 + homologues in the inlet gas (e.g., ethanol from ethane, etc.), and/or accumulation of terminal exometabolites from the producer’s metabolism, such as organic acids (e.g., formic, acetic, fumaric, oxalic, propionic acids, etc.).

The linear increase in the mean projected “shadow” area of all morphotypes [by 11.2–13.8 % over 9 (h)] correlates with an increase in cell radius (from 0.734 to 0.774 μm for *monococci* and from 0.602 to 0.634 μm for *diplococci*), intracellular volume (by 16.9–19.5 %), and surface area (by 11.0–14.2 %), accompanied by a simultaneous decrease in the *A/V* ratio (by 4.1–5.1 %). These changes might be interpreted as an adaptation to cultivation conditions: increasing cell volume during the G1 phase allows the accumulation of “critical mass” for initiating the S-phase, while the expansion of the periplasmic membrane area enhances the accommodation of pMMO (periplasmic methane monooxygenase), and the folding of the cytoplasmic membrane accommodates mMMO (membrane-bound form). The decrease in *A/V* indicates a shift toward volume-oriented growth, which may improve methane oxidation efficiency under substrate-limiting conditions. The constant ratio of areas *S*_*diplococci*_ / *S*_*monococci*_ 1.45 confirms that the transition from *monococci* to *diplococci* occurs through the redistribution of the existing volume, without a significant gain of new biomass during the M-phase.

The obtained results demonstrate different patterns in the size distribution of bacterial cells belonging to different morphotypes. The “shadow” area distributions reflect population heterogeneity: a normal distribution for *tetracocci* (*p* > 0.05 according to the Shapiro-Wilk test) indicates synchrony, while deviations for *monococci* and *diplococci* (*p* < 0.05) suggest the presence of subpopulations and asynchronous division. This aligns with metabolomic studies where stress (e.g., from exometabolite accumulation) induces heterogeneity in the TCA cycle and glutathione metabolism (Hartl et al., 2020). In the context of gaprin (i.e., bacterial SCP) production on natural gas, monitoring morphological composition of the bacterial consortium using AI enables the early prediction of productivity deviations, which is critical for a large-scale SCP production.

Despite its advantages, the method has limitations: dependence on image quality (resolution, focus, contrast, etc.) and the assumption of spherical cell geometry, which may not account for shape variations in the polysaccharide capsule. Moreover, analysis of a pure culture does not fully reflect the dynamics within the consortium with satellites, where interspecies interactions can modulate the cell cycle. The analysis of prepared sample images cannot be accurately linked to cell concentration in working volume of the bioreactor. This is due to the irreproducibility of biomass concentration and the formation of liquid volume between the slide and coverslip on the microscopic preparation. To overcome this limitation, a flow-through chamber with a known volume must be used.

Overall, integrating AI into population dynamics monitoring opens prospects for real-time process control in SCP biotechnology, contributing to enhanced efficiency of gaprin production and its competitiveness as a feed protein.

## 5 Conclusion

The trained neural network has successfully determined cell morphotypes, fractional composition and dynamics of the mixed bacterial culture based on the methanotroph *Methylococcus capsulatus* KN2. This combined analytical approach confirms the technical feasibility of developing real-time monitoring systems to optimize the cultivation process and control the quality of the bacterial SCP derived from methane.

The key results:

- The trained convolutional neural network YOLO11x-seg was trained on 250 images of the industrial methanotrophic consortium based on *Methylococcus capsulatus* KN2 with satellite bacteria and successfully identified and segmented 9 cell morphotypes, in a total of 50,410 objects.
- Under continuous cultivation conditions in several experiments [μ = 0.15–0.25 (1/h)], the population structure of the consortium was characterized by the dominance of the producing strain *M. capsulatus* KN2–88.5% of the total number of cells, with the fraction of heterotrophic satellite bacteria being 11.5%.
- Within the producer population, the distribution of morphotypes was:
  ○ *monococci*–33.7%,
  ○ *diplococci*–61.9%,
  ○ *tetracocci*–4.4%. The *tetracocci* are considered as a sub-population of *diplococci* fraction.
- During substrate-unlimited batch cultivation [*t* = 9 (h), μ_*max*_ = 0.223 (1/h), *t_d_* = 3.11 (h), three replicates, four time points] of pure *Methylococcus capsulatus* KN2 culture in gas tight shaking flasks 277 experimental images were collected, from which 15,124 objects belonging to *mono*-, *diplo*-, *tetracocci* morphotype classes were identified.
- During substrate-unlimited batch cultivation of pure *Methylococcus capsulatus* KN2 culture, the dynamics of morphotypes was observed:
  ○ the fraction of *monococci* has decreased from 35.4 to 28.8%,
  ○ the fraction of *diplococci* has increased from 61.0 to 68.6%,
  ○ there is the significant negative correlation between content of *diplococci* and *monococci* (Pearson *r* = −0.996, *p* = 0.004).
  ○ the fractional content of *tetracocci* is random, and it does not statistically link to the fractional content of *diplococci.*
- There is a dynamic change in the morphology of *monococcal* cells of pure *Methylococcus capsulatus* KN2 culture during the substrate-unlimited batch cultivation process:
  ○ the average cell radius has increased from 0.734 to 0.773 [μm];
  ○ the average intracellular volume has increased from 1.659 to 1.941 [μm^3^];
  ○ the average periplasmic surface area has increased from 6.776 to 7.524 [μm^2^];
  ○ the average surface/volume ratio has decreased from 4.085 to 3.877 [μm^2^/μm^3^].
- The mean ratio of object areas *diplococci/monococci* = 1.45 at any time point of batch cultivation.
- Cell cycle analysis revealed an extension of the M-phase from 71.6 to 78.4% of the total cycle time, which is interpreted as an early sign of substrate limitation (CH_4_ or O_2_), potentially not captured by standard process parameters.
- The proposed AI approach provides quantitative monitoring of culture quality in near real-time and can be used to predict limiting growth factors and optimize SCP yield in industrial bioreactors.

## 6 Prospective

The results obtained in this study open prospective for further methodological development of AI-assisted bioprocess monitoring: 1. precise determination of the cell radius within *diplococci* is required to improve morphometric accuracy and reduce uncertainty in volume-based biomass estimations, particularly for dividing cells.

2. high-resolution biomass concentration (*C_x_*) measurements should be integrated with image-based population analysis to enable accurate recalculation of changes in the maximal specific growth rate (μ_*max*_), biomass doubling time (*t_d_*), and associated shifts in the fractional composition of the culture.

3. direct correlation between image-derived cell counts and absolute cell concentrations in the bioreactor working volume will allow quantitative scaling of microscopy-based observations to process-level parameters.

## Data Availability

The experimental data and MATLAB scripts are available at https://github.com/Maks3axar/MethaVision.

## Footnotes

1
https://app.cvat.ai/

2
https://docs.ultralytics.com/models/yolo11/
