# Supplementary material for: Population dynamics analysis of an industrial methanotrophic consortium based on Methylococcus capsulatus KN2 using AI technologies
Source: Front Microbiol. 2026 Jun 17;17:1831666. doi: 10.3389/fmicb.2026.1831666 (PMC13322215; doi:10.3389/fmicb.2026.1831666)
Supplement: Supplementary file 1 [file Supplementary_file_1.docx]

Supplementary Material

# Supplementary Data

The experimental data and MATLAB scripts are available at <https://github.com/Maks3axar/MethaVision>

Derivation of equation (7):

1. Distribution of cell ages: In an exponentially growing asynchronous population $N\left( t \right)=N_{0}e^{\left( \mu\cdot t \right)}$ the probability density of a cell having age $a$ (from 0 to $t_{d}$) is given by:

$$p\left(a\right)=\frac{\ln2}{t_{d}}\cdot2^{\left[\left(t_{d}-a\right)/t_{d}\right]}$$

This follows from the fact that the number of cells of age $a$ proportional to $2^{\left( -a/t_{d} \right)}$ (younger cells predominate because the population doubles every *t_d_*).

1. Normalization check:

$\int_{0}^{t_{d}} p\left(a\right)da=1$( total probability)

1. Fraction in M‑phase: M is the last phase (age from $t_{d}-t_{M}$ to $t_{d}$)

$$f_{M}=\int_{t_{d}-t_{M}}^{t_{d}} p\left(a\right)da$$

Substitution and integration yields:

$$f_{M}=2^{\left[t_{M}/t_{d}\right]}-1$$

Calculation of geometric dimensions of cell morphotypes monococci and diplococci from «shadow» area.

| *monococci* | *diplococci* |
| --- | --- |
| **Assumption**: *Monococci is a perfect sphere.* | **Assumption**: *diplococci* can be represented as two equal overlapping perfect spherical *monococci* with identical cell radii (*r_1_* = *r_2_*). The cell radius of *diplococci* can be taken from calculations of the cell radius of *monococci* (*i.e.* *r_1_* = *r_2_ = 0.8×r_monococci_*). The coefficient 0.9 was estimated empirically from visual observation during the annotation of micrographs in the neural network training process. |
| **It is known that**:   - *S_monococci_* – experimentally measured "shadow" area of *monococci* (*area_μm2;* Table 4). | **It is known that**:   - *S_monococci_* – experimentally measured "shadow" area of *monococci* (*area_μm2;* Table 4). - *S_diplococci_* – experimentally measured "shadow" area *of diplococci* (*area_μm2;* Table 4). |
| **Task** - calculate:   - cell radius (*r_monococci_*); - intracellular volume (*V_monococci_*); - periplasmic surface area (*A_monococci_*); - *A/V* ratio for *monococci*. | **Task -** calculate:   - cell radius (*r_monococci_*); - *d* – distance between the centers of two overlapping cells ($0\leq d\leq2r$) - *h* – height of the spherical segment - intracellular volume (*V_diplococci_*); - periplasmic surface area (*A_diplococci_*); - *A/V* ratio for *diplococci*. |
| **Solution**:  cell radius of *monococci*: $r_{monococci}=\sqrt{\frac{S_{monococci}}{\pi}}$  [μm]  intracellular volume *monococci*: $V_{monococci}=\frac{4}{3}\pi r_{monococci}^{3}$  [μm^3^]  periplasmic surface area *monococci*: $A_{monococci}=4\pi r_{monococci}^{2}$  [μm^2^]  Surface‑to‑volume ratio of *monococci*: $A/V=\frac{A_{monococci}}{V_{monococci}}$  [μm^2^/μm^3^] | **Solution**:  cell radius of *diplococci*: $r_{diplococci}=0.8r_{monococci}=0.8\sqrt{\frac{S_{monococci}}{\pi}}$  [μm]  Equation for the shadow area of *diplococci* as a function of cell radius and the distance (*d*) between the centers of two overlapping cells: $\begin{matrix} S_{diplococci}^{*}=2r_{diplococci}^{2}\left(\pi-\arccos\left(\frac{d}{2r_{diplococci}}\right)\right)+ \\ +d\sqrt{4r_{diplococci}^{2}-d^{2}} \end{matrix}$   By iterating d within the range 0 ≤ *d* ≤ *2r*, it is possible to find the value of *d* at which the equality holds: $f=S_{diplococci}^{*}\left(d\right)-S_{diplococci}=0$   To calculate the intracellular volume (*V_diplococci_*) and the periplasmic surface area (*A_diplococci_*) of *diplococci*, the volume of each sphere (of the two *monococci*) must be adjusted by subtracting the spherical segment that extends beyond the plane of sphere overlap.  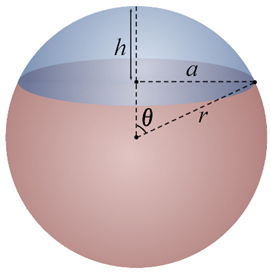  Height of the spherical segment (*h*): $h=r_{diplococci}-\frac{d}{2}$   The radius of the base of the spherical segment (*a*) is calculated using the Pythagorean theorem: $a=\sqrt{r_{diplococci}^{2}-\left(r_{diplococci}-h\right)^{2}}$   The volume of the spherical segment is equal to: $V_{cap}=\frac{\pi h}{6}\left(3a^{2}+h^{2}\right)$  [μm^3^]  Intracellular volume of *diplococci*: $\begin{matrix} V_{diplococci}=2V_{monococci}-2V_{cap}= \\ =2\left[\left(\frac{4}{3}\pi r_{diplococci}^{3}\right)-\left(\pi h^{2}\frac{3r_{diplococci}-h}{3}\right)\right] \end{matrix}$   Surface area of the spherical segment: $A_{cap}=\pi\left(a^{2}+h^{2}\right)$  [μm^2^]  Periplasmic surface area of *diplococci*: $\begin{matrix} A_{diplococci}=2A_{monococci}-2A_{cap}= \\ =2\left[\left(4\pi r_{diplococci}^{2}\right)-\left(2\pi r_{diplococci}h\right)\right] \end{matrix}$   Surface‑to‑volume ratio of *diplococci*: $A/V=\frac{A_{diplococci}}{V_{diplococci}}$  [μm^2^/μm^3^]  MATLAB:  % Experimental data  S_mono = 1.694; % projected area of monococcus, [μm²]  S_diplo = 2.424; % projected area of diplococcus, [μm²]  % 1. Cell radius  r = sqrt(S_mono / pi) * 0.8; % [μm]  fprintf('Cell radius r = %.5f μm\n', r);  % 2. Solving for the distance d between centers  S_diplo_func = @(d) 2*r^2*(pi - acos(d/(2*r))) + d*sqrt(4*r^2 - d^2);  f = @(d) S_diplo_func(d) - S_diplo;  d = fzero(f, 0.9); % exact solution  fprintf('Distance between centers d = %.5f μm (ξ = %.3f)\n', d, d/(2*r));  % 3. Volume and surface of diplococcus (two intersecting spheres)  h = r - d/2; % height of the spherical segment, [μm]  V_cap = pi * h^2 * (3*r - h) / 3; % [μm³]  V_sphere = 4/3 * pi * r^3; % [μm³]  V_diplo = 2*V_sphere - 2*V_cap; % [μm³]  A_cap = 2 * pi * r * h; % [μm²]  A_sphere = 4 * pi * r^2; % [μm²]  A_diplo = 2*A_sphere - 2*A_cap; % [μm²]  fprintf('Intracellular volume V_diplo = %.5f μm³\n', V_diplo);  fprintf('Surface area A_diplo = %.5f μm²\n', A_diplo);  fprintf('A/V ratio = %.4f μm⁻¹\n', A_diplo/V_diplo);  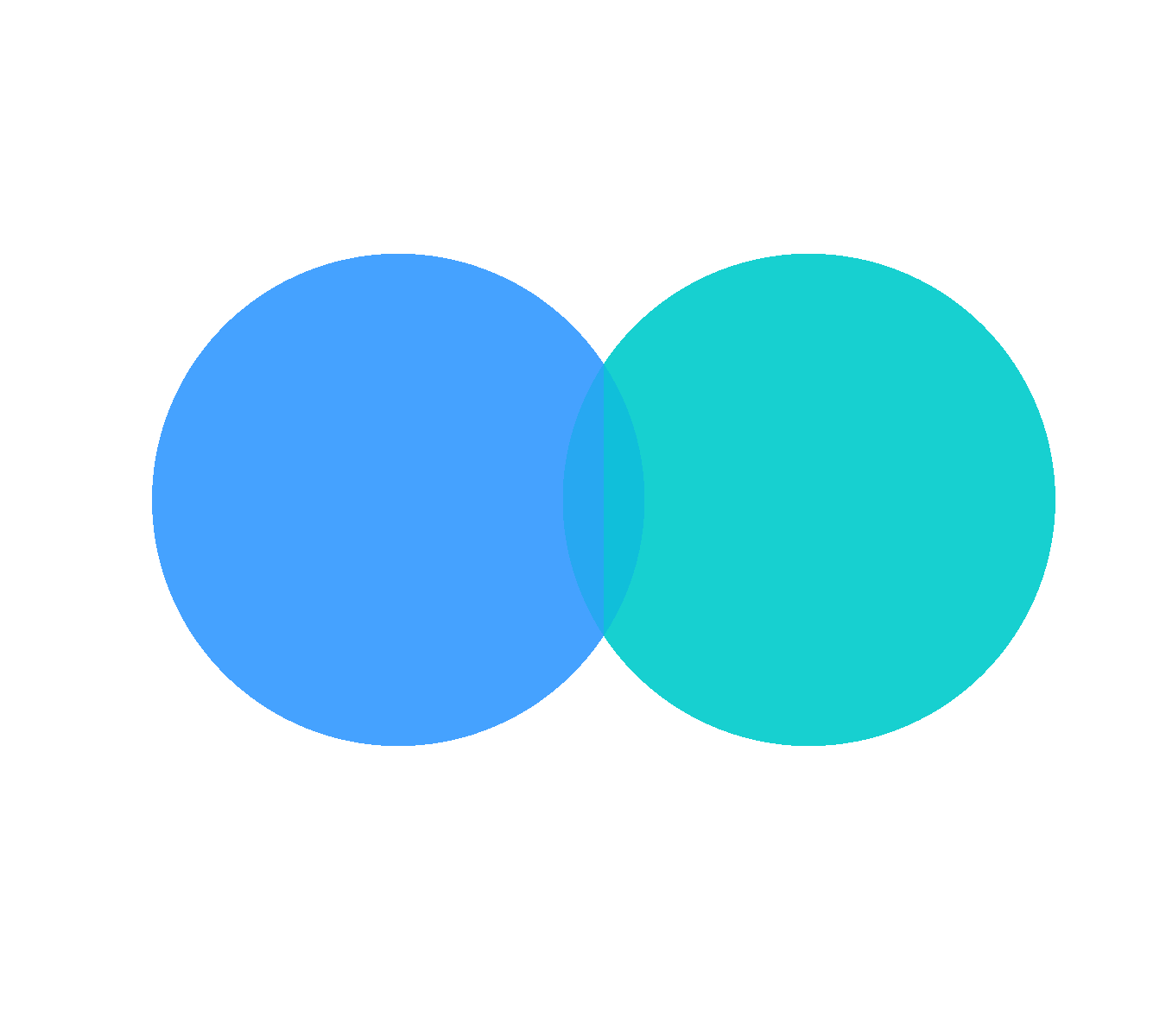  Visualization of the calculated geometric shape of *diplococci* based on the assumptions and calculations presented above, highlighting the lens of sphere intersection. The volume and area of this lens have been subtracted to adjust the geometric dimensions of *diplococci* (i.e., intracellular volume, periplasmic surface area). |

# Supplementary Figures and Tables

## Supplementary Tables

Table S1

Morphotypes of satellite bacteria selected for training the neural network. Scale bar: 5 µm.

| Morphotype | Shape and size | Example |
| --- | --- | --- |
| bacilli-like | very large and thick rods over 10 µm long and over 2 µm wide, spore-forming rods | 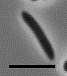 |
| long_rods | long, large rods 3-10 µm long, 1-2 µm wide | 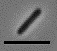 |
| small_rods | small rods 1-3 µm long | 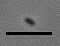 |
| thin_rods | thin rods 4-6 µm long, no more than 1 µm wide | 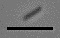 |
| double_cell | double (dividing) rods | 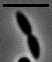 |
| acr_cell | arcuate cells of any size | 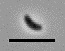 |

Table S2

Methods of analysis of species and fractional composition of a bacterial consortium.

| Method | Pros & Cons |
| --- | --- |
| Microbiology – classical quantitative colony counting on solid agar medium | Low data discretization, weak qualitative and quantitative analysis |
| Molecular genotyping based on 16S rRNA | Low data discretization, expensive, difficult to perform accurate quantitative analysis |
| Particle counting and sizing by flow cytometry | Instruments for measuring ~1 μm particles are expensive, difficult to calibrate by size and morphotype, does not distinguish cell morphotypes, generates large data volume |
| Particle counting and sizing by Coulter counter | Low accuracy in morphotype recognition of cells of similar size, generates large data volume |
| Microscopy | Low data discretization, low throughput, inexpensive, generates small data volume – high probability of statistical error, result depends on an operator |

Table S3

Statistical analysis of the normality of the distribution of calculated "shadow" areas for the cell morphotypes of *Methylococcus capsulatus* KN2 cultivated under non-sterile conditions in continuous mode in bioreactor in the combined dataset (Training and Validation datasets; **Table 2**).

|  | *n* | Shapiro-Wilk Test | D'Agostino's χ²  Test | Kolmogorov-Smirnov test | Skewness | Kurtosis |
| --- | --- | --- | --- | --- | --- | --- |
| *monococci* | 27 643 | W = 0.9854,  p = 2.6010e-36 | χ² = 935.5399,  p = 7.0810e-204 | D = 0.0301,  p = 3.0303e-12 | 0.5305 | 1.0517 |
| *diplococci* | 15 003 | W = 0.9728,  p = 1.7418e-56 | χ² = 2653.3410,  p = 0.0000e+00 | D = 0.0434,  p = 1.3672e-45 | 0.7376 | 1.1259 |
| *tetracocci* | 1 977 | W = 0.9595,  p = 4.5750e-23 | χ² = 122.9269,  p = 2.0266e-27 | D = 0.0751,  p = 3.7602e-10 | 0.6655 | 0.0208 |

Table S4

Pairwise comparisons using the Mann-Whitney U test with Bonferroni correction. Number of comparisons: 3, adjusted α (Bonferroni): 0.016667.

| Compared groups | N (Sample 1 / Sample 2) | U-statistics | p-value (original) | p-value (with Bonferroni correction) | Significance (for α = 0.016667) | Effect size (r) | Effect interpretation |
| --- | --- | --- | --- | --- | --- | --- | --- |
| *Diplococci*  vs  *Tetracocci* | 27643 / 1977 | 12 067 169.0 | < 0.000001 | < 0.000001 | Yes (p < 0.016667) | 0.558 | Large |
| *Diplococci*  vs  *Monococci* | 27643 / 15003 | 348 373 028.0 | < 0.000001 | < 0.000001 | Yes (p < 0.016667) | 0.680 | Large |
| *Tetracocci*  vs  *Monococci* | 1977 / 15003 | 28 139 887.0 | < 0.000001 | < 0.000001 | Yes (p < 0.016667) | 0.897 | Large |


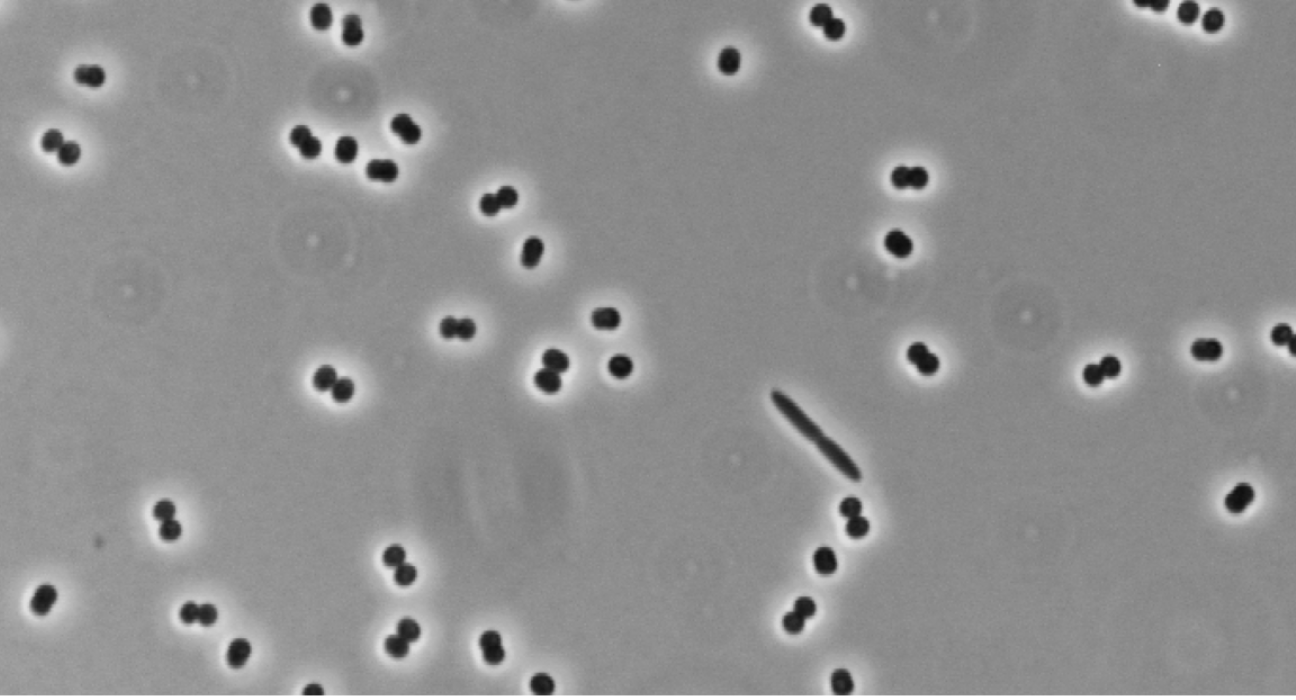


**A**


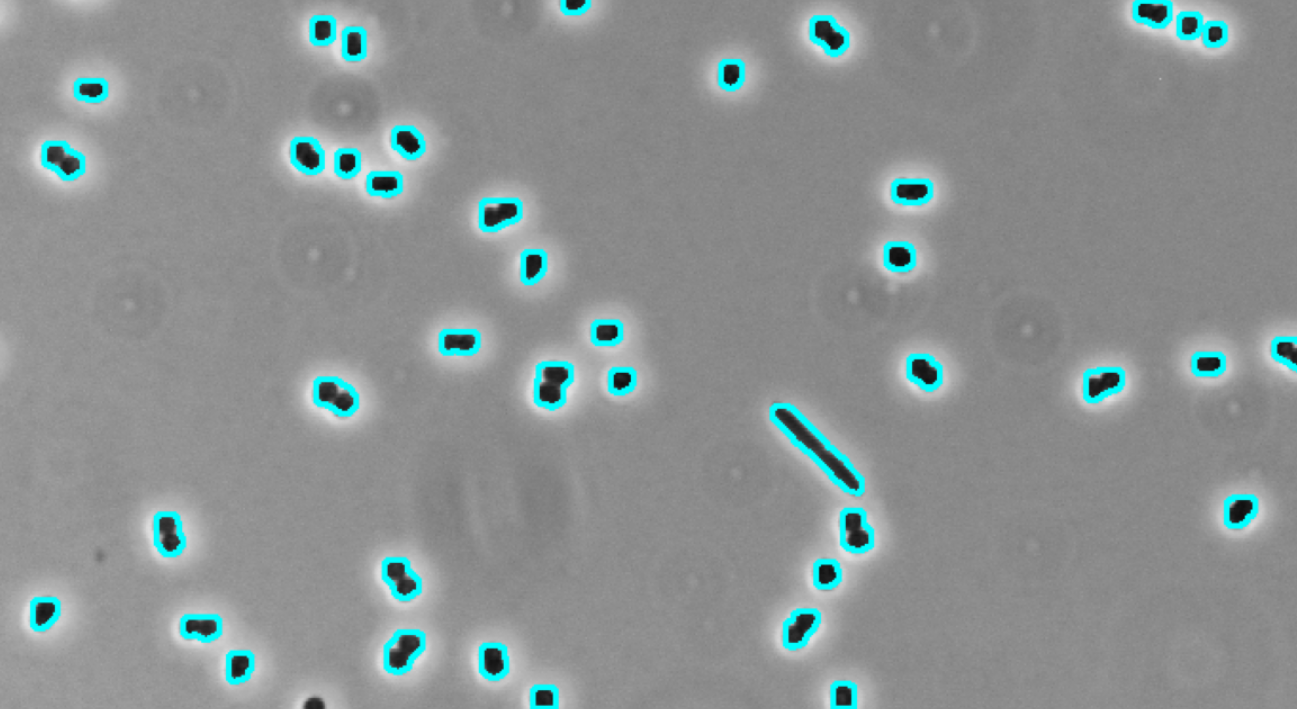


**B**

**Figure S1**. An example of close-up view on (A) an original image and (B) its segmentation after cells identification. This figure is the supplementary material to the **Figure 7**.

Table S5

Examples of close-up views on segmentation masks for some objects from Figure S1.

| Class | Object |
| --- | --- |
| *diplococci* | 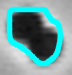 |
| *diplococci* | 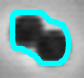 |
| *diplococci* | 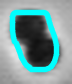 |
| *diplococci* | 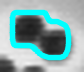 |
| *diplococci* | 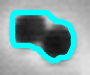 |
| *monococci* | 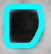 |
| *monococci* | 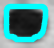 |
| *monococci* | 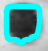 |
| *bacilli-like* | 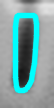 |
| *bacilli-like* | 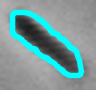 |

# ****Supplement 3****

****Development and training of a neural network for cell morphotype recognition****

## ****Model architecture and loss function****

**Cell segmentation and detection were performed using the YOLO11x-seg architecture with a custom configuration, including activation of the P2 layer (resolution 160 ×160 pixels). The P2 layer ensures the capture of fine-grained features early in processing, which is critical for detecting *monococci* with a size of 14 ×14 pixels**^[[1]](#footnote-1)^**.**

**The standard CIoU (Complete IoU) function proved ineffective for solving the problem of segmenting small objects (*monococci* are only 14 ×14 pixels in micrographs), because, for very small objects, the distance between the center of the predicted and actual bounding boxes is normalized to the diagonal of the smallest enclosing rectangle. At this scale, even a small error of 2-3 pixels can result in the same loss as boxes with significantly different shapes, slowing convergence and reducing accuracy.**

**To optimize the regression of bounding boxes of small objects, a combined *Focaler-MPDIoU* loss function was used:**

$$L_{focaler-MPDIoU}=1-\mathrm{Io}U_{f\mathrm{ocaler}}+\frac{d_{1}^{2}}{w^{2}+h^{2}}+\frac{d_{2}^{2}}{w^{2}+h^{2}}$$

The *Focaler-MPDIoU* function combines: *MPDIoU* (Minimum Point Distance IoU), which directly minimizes the distance between the corner points of the predicted and actual boxes; *Focaler*, which adds a focusing mechanism for complex examples (such as small bacteria), dynamically adjusting the sample weights. Where ${IoU}_{focaler}$ is a focused version of IoU (Intersection over Union), dynamically adjusting the weights of complex examples, $d_{1}$ and $d_{2}$ are the distances between the corresponding corner points of the predicted and actual boxes, and *w* and *h* are the width and height of the minimum bounding rectangle. This approach overcomes the fundamental limitation of standard CIoU for small objects, where a localization error of 2-3 pixels becomes barely noticeable.

## ****Weighted Loss Function to Decrease Class Imbalance****

To compensate for class imbalance, a weighted loss function was used. Class weights were calculated as the inverse of the occurrence frequencies:

$$w_{c}=N_{total}/\left( K\cdot N_{c} \right)$$

where $N_{total}$ = 39 900 – is the total number of annotated objects in the training set, $K=9$ – is the number of morphotype classes, $N_{c}$​ – the number of objects of class *c* in the training dataset. To prevent training instability, the weights were limited to a maximum value of 30 (clipping).

The weights were restricted at a maximum value of 30 (clipping) to prevent training instability. The final weights were: *diplococci* 0.20; *monococci* 0.38; *small_rods* 2.39; *tetracocci* 2.55; *long_rods* 3.85; *bacilli-like* 7.02; *thin_rod* 8.79; *double_cells* 25.04; *arc_cells* 30.00 (clipping).

## Activation of the P2 layer

To improve detection of small objects (*monococci* with size 14**×**14 pixels), the P2 branch of the YOLO architecture was activated. The P2 layer operates at a resolution of 160**×**160 pixels and ensures the capture of fine-grained features early in processing. This allowed the model to identify small objects that are missed when using only standard P3/P4/P5 layers.

## Two-stage training

To reduce class imbalance, a two-stage training strategy was used:

Stage 1 (pre-training on a balanced subset):

- A balanced subset of approximately 2 000 objects was created (about 200-220 objects of each class, including synthetic *tetracocci*).
- Training: 50 epochs, learning rate 1×10^−3^, AdamW optimizer, batch size = 8, weight decay = 5×10^−4^.
- Augmentation: reflections, shifts, scaling, HSV variations.

Stage 2 (additional training on the full dataset):

- The model was additionally trained on the full training set (200 original images + 12 synthetic images, 39 900 objects) for up to 250 epochs.
- Early stopping (patience = 50 epochs) based on mAP@0.5:0.95 monitoring on the validation set.
- Advanced augmentation: mosaic (1.0), mix-up (0.5).

## Training hyperparameters

Training was performed with the following hyperparameters:

| **Parameter** | **Value** | **Justification for use** |
| --- | --- | --- |
| Epochs | 250 | With early stopping at patience = 50 |
| Image size | 1024×1024 | Preservation of small object details |
| Batch size | 8 | GPU memory limitation |
| Optimizer | AdamW | Automatic learning rate selection |
| Weight decay | 5×10⁻⁴ | Regularization |
| Augmentation (mosaic, mixup) | 1.0; 0.5 | Increasing data diversity |

## Learning Outcomes

**On the validation set (50 images, 10 509 objects), the model achieved:**

**mAP@0.5:0.95 = 0.52.**

**Weighted precision = 0.87, recall = 0.85, F1-score = 0.86.**

**After implementing the P2-layer and *Focaler-MPDIoU*, the following metrics were obtained.:**

| Class | Precision | Recall | F1-score | mAP50-95 |
| --- | --- | --- | --- | --- |
| diplococci | 0.876 | 0.906 | 0.890 | 0.514 |
| monococci | 0.792 | 0.718 | 0.753 | 0.394 |
| tetracocci | 0.614 | 0.692 | 0.651 | 0.520 |
| **Weighted average** | **0.87** | **0.85** | **0.86** | - |

**All calculations were performed on an NVIDIA RTX A6000 GPU (49 GB).**

1. [https://www.sciencedirect.com/science/article/abs/pii/S0262885625004822?via%3Dihub#preview-section-cited-by](https://www.sciencedirect.com/science/article/abs/pii/S0262885625004822?via%3Dihub%23preview-section-cited-by) [↑](#footnote-ref-1)
